# Elevated PRDM13 Disrupts Photoreceptor Function and Survival in the Mammalian Retina

**DOI:** 10.1167/iovs.66.11.38

**Published:** 2025-08-18

**Authors:** Emily R. Nettesheim, Ashley A. Rowe, Tiffany Yee, Ahmed Alshaikhsalama, Tyler Cepica, Vijaya Dutt, Samita S. Virani, Glen Wickersham, Vinit B. Mahajan, Kent W. Small, Katherine J. Wert

**Affiliations:** 1 Department of Ophthalmology, University of Texas Southwestern Medical Center, Dallas, Texas, United States; 2 Molecular Surgery Laboratory, Byers Eye Institute, Department of Ophthalmology, Stanford University, Palo Alto, California, United States; 3 Veterans Affairs Palo Alto Health Care System, Palo Alto, California, United States; 4 Macula and Retina Institute, Molecular Insight Research Foundation, Glendale, California, United States; 5 Department of Molecular Biology, University of Texas Southwestern Medical Center, Dallas, Texas, United States; 6 Peter O'Donnell Jr. Brain Institute, University of Texas Southwestern Medical Center, Dallas, Texas, United States; 7 Hamon Center for Regenerative Science and Medicine, University of Texas Southwestern Medical Center, Dallas, Texas, United States

**Keywords:** retinal degeneration, photoreceptor degeneration, PRDM13, NR2E3, retinal dystrophy

## Abstract

**Purpose:**

Retinal degeneration is a common cause of blindness, but there is a gap in our understanding of the molecular mechanisms causing degeneration. Dysregulated PRDM13 has been linked to retinal dystrophy, indicating a role for PRDM13 in the retina. PRDM13 knockout studies have shown that PRDM13 specifies amacrine cell fates, but no studies have shown the phenotypic and mechanistic outcomes of its elevated activity in the retina.

**Methods:**

We developed a mouse model to induce aberrant *PRDM13* expression in a controlled, time-dependent manner. ERG and histological analyses were performed to determine whether elevated *PRDM13* impacted the health and function of the retina. RNA sequencing of retinas with and without elevated *PRDM13* defined transcriptional changes in response to PRDM13 activity. Differentially expressed genes were validated by quantitative PCR and western blot.

**Results:**

We found that elevated *PRDM13* decreases photoreceptor function and survival. Depletion of elevated *PRDM13* halted this degenerative phenotype and restored some photoreceptor function. We further uncovered that elevated *PRDM13* alters the expression of markers for amacrine subpopulations. Transcriptomic profiling revealed that elevated *PRDM13* deregulates genes involved in retinal development, phototransduction, and photoreceptor health. We found downregulation of *Prdm1*—a photoreceptor marker—and *Nr2e3*—a key regulator of photoreceptor specification also implicated in retinal dystrophies—as well as effects on NR2E3’s direct and indirect targets.

**Conclusions:**

These findings establish a critical role for PRDM13 in retinal health and provide a new model for elucidating the impact of PRDM13 on photoreceptor maturation, maintenance, and function during retinal development.

Retinal degenerations are among the most common causes of blindness in humans, including macular degeneration and inherited retinal dystrophies. Unfortunately, there continues to be a gap in our understanding of the basic molecular mechanisms leading to retinal degeneration, which in turn has impeded progress toward more effective treatments. Photoreceptors are essential retinal neurons that respond to photons of light and initiate the signaling cascades that lead to cortical processing and the perception of vision. If these postmitotic neurons are lost, they cannot be regenerated. Thus, it is critical to understand the mechanisms of photoreceptor degeneration to develop new therapeutic strategies to promote the maturation, maintenance, and protection of photoreceptors.

The positive regulatory domain member (PRDM) family has been shown to drive and maintain cell states in a wide range of tissues and cell types.^1^ One member of the PRDM family, PRDM1, is known to promote specification of photoreceptors during retinal development.^2^^–^^4^ Another member of this family, PRDM13, has been associated with the development of the neural tube and retina, and it has been implicated in the retinal disease North Carolina macular dystrophy (OMIM #136550).^5^^–^^8^ However, no direct evidence has been shown in patients with North Carolina macular dystrophy for dysregulation of PRDM13. To date, PRDM13 has been best characterized during early neural tube development, where it is known to act within a negative feedback loop to repress its own expression through primary repression of PTF1a.^9^^–^^11^ PRDM13 has also been found to act as a transcriptional repressor of TLX1/3 through the inhibition of ASCL1 activity, thereby promoting the inhibitory neuron fate at the expense of excitatory neurons.^10^^–^^12^ Within the retina, deletion of exons 2 and 3 of *Prdm13* generated viable mice with a reduction in GABAergic and glycinergic amacrine cells by postnatal day (P)14.^13^ Another mouse with green fluorescent protein knocked into the first exon of *Prdm13* and followed by a stop codon was homozygous neonatal lethal, but viable in the heterozygous state.^12^^,^^14^ Studies in this mouse model revealed no changes in retinal development through embryonic day (E)17.5.^14^ However, crossing this mouse line with one carrying a frameshift in *Prdm13*, which resulted in significantly reduced *Prdm13* expression, prevented lethality and revealed a loss of the early B-cell factor 3–positive amacrine cell subtype.^14^

Although studies have investigated the loss of PRDM13 in the retina, little is known about the impact of elevated PRDM13, which is believed to be causal for North Carolina macular dystrophy.^7^ In a mouse model of *Prdm13* deficiency, forced overexpression of *Prdm13* in the retina at P0 resulted in a detrimental effect on photoreceptor survival at P6.^13^ This result was recapitulated in vitro, as mouse retinal explants overexpressing *Prdm13* via transient transfection failed to form photoreceptors.^14^ In *Drosophila*, knockdown of the *PRDM13* orthologue resulted in no detectable eye phenotype, but overexpression of the *PRDM13* orthologue led to a loss of the imaginal eye antennae disc, indicative of abnormal retinal development.^6^ Although these prior studies suggest a role for PRDM13 in photoreceptor maintenance, no studies have further investigated the impact of elevated *PRDM13* expression on the retina, and the regulatory targets of PRDM13 within the retina remain unknown.

In this study, we developed a mouse model to induce aberrant *PRDM13* expression in a controlled, time-dependent manner. We found that systemic overexpression of *PRDM13* can act through its known regulatory feedback loop during development and that it affects the retina by reducing photoreceptor function and survival. Depletion of elevated *PRDM13* halted this degenerative phenotype and restored some photoreceptor function after immune responses to stress dissipated. Because PRDM13 is believed to act as a transcriptional repressor,^9^^–^^12^ transcriptomic profiling was performed on neural retina samples, and this work revealed that aberrant *PRDM13* expression specifically dysregulates genes involved in retinal development, phototransduction, and photoreceptor health. Markedly, we found that *PRDM13* expression downregulated the photoreceptor marker *Prdm1* as well as *Nr2e3*—a key regulator of photoreceptor specification also implicated in retinal dystrophies—and affected NR2E3’s direct and indirect targets.^15^^,^^16^ Additionally, we found that elevated *PRDM13* alters multiple genes important for amacrine cell identity and function, including *Robo3* and *Mef2c*, markers for amacrine cell subtypes not previously associated with PRDM13.^17^ These findings establish a critical role for PRDM13 in retinal health and provide a new model for further elucidating the action of PRDM13 in photoreceptor maturation, maintenance, and function during mammalian retinal development.

## Methods

### Mouse Lines and Husbandry

Mice were bred and maintained in animal facilities at the University of Texas Southwestern Medical Center. Animals were kept on a standard light–dark cycle (12 hour–12 hour). Food and water were available ad libitum throughout the experiment. For all experiments, *PRDM13*-inducible mice carrying one copy of the knock-in construct—herein referred to as PRDM13-OE mice—were used along with wildtype littermates. This approximately 6 kilobase knock-in contained the EF-1α ubiquitous promoter driving reverse tetracycline-controlled transactivator expression as well as the TRE3G promoter driving 3xHA-*PRDM13* expression*.* The knock-in construct was inserted into the *Rosa26* safe harbor locus in the C57BL/6J background (Jackson Laboratory, Bar Harbor, ME, USA; RRID: MSR_JAX:000664) using TARGATT technology (Applied StemCell, Milpitas, CA, USA). Mice were screened, and tested negative, for common retinal degenerative mutations (*Rd1*, *Rd8*, and *Gnat2*) using PCR.^18^ Our study examined male and female animals, and similar findings are reported for both sexes.

### Administration of Doxycycline (dox)

Mice were provided 2 mg/mL dox hyclate (Sigma-Aldrich, St. Louis, MO, USA; D9891-100G) with 10% sucrose (Sigma-Aldrich, S0389-1KG) orally via drinking water. Treated water was provided ad libitum in red-tinted water bottles for between 1 and 10 days depending on the experimental group. For treatment durations longer than 3 days, water was changed to avoid microbial growth and ensure efficacy following approved institutional guidelines.

### Histology and Nuclei Quantification

Eyes were collected and sectioned following previously published methods.^19^^,^^20^ Nuclei were quantified from retinal sections in both the outer nuclear layer (ONL) and inner nuclear layer (INL). For quantification, the area between the optic nerve head and the ciliary body was divided into three sections on either side. Four columns of nuclei were counted for each region per section to get average nuclei counts across the ONL and INL for each of the experimental and control groups. Only eyes with no artifacts from sectioning, that is, tissue tearing, were used for nuclei counts.

### Immunohistochemistry

Mouse eyes were fixed, cryopreserved, and sectioned for immunohistochemistry as previously described.^20^ Slides were imaged using a Leica SP8 laser scanning confocal microscope with either a 25× water or 63× oil immersion objective lens. For retinal flat mounts, mouse eyes were fixed, dissected, then stained following the same methods as the sagittal sections, then imaged with the 25× water immersion lens in both the central (around the optic nerve head) and peripheral regions of the retina. At least one section was imaged for each of three biological replicates. Antibodies can be found in Supplementary Table S2.

### Optical Coherence Tomography (OCT)

OCT images were collected using the Spectralis OCT Scanning Laser Ophthalmoscope (Heidelberg Engineering, Franklin, MA, USA) following previously published methods.^19^^,^^20^ All images were collected using a 55° wide lens. OCT images were taken at a plane that transected the optic nerve head and a composite of 100 frames was collected.

### Total Retina Thickness (TRT) Measurements

TRT measurements were collected from the OCT images using the measurement tool in the Heidelberg Eye Explorer Software on the micron (µm) setting. Starting at the optic nerve head, designated as 0 mm on the image, measurements were collected at 1, 2, 3, and 4 mm on either side of our designated 0 point on the image for a total of eight measurements. TRT was measured from the top of the retinal nerve fiber layer to the bottom of the RPE.

### ERG

Scotopic and photopic ERG recordings were collected on the Celeris ERG system from Diagnosys (Lowell, MA, USA) following previously published methods.^19^ Mice were exposed to two scotopic light settings (0.01 and 1.0 cd•s/m^2^), a 10-minute white light adaptation, followed by two photopic light settings (10.0 cd•s/m^2^ and a 10 Hz flicker) with an average of at least 10 sweeps per eye. Pattern ERG (PERG) was performed according to previously published methods.^19^ The PERG stimulator was placed on the right eye and a corneal ground reference on the left eye, with additional ground electrodes placed on the forehead, cheek, and thigh. Three hundred sweeps were averaged per eye.

### Quantitative PCR (qPCR)

RNA was extracted from tissue samples following the standard protocol of the Qiagen RNeasy Mini Kit (Qiagen, Germantown, MD, USA; 74106); up to 900 ng of purified RNA for each sample was then converted to cDNA following the standard protocol for the high-capacity RNA-to-cDNA kit (Applied Biosystems, Waltham, MA, USA; 4387406). qPCR reactions used SYBR green PCR master mix (Applied Biosystems, 4309155) and 10 ng of cDNA per well. Samples were run on the QuantStudio FLEX6 System (Applied Biosystems, 4485691), and data were reported as delta CT values, with beta-actin or *U36b4* used as the housekeeping gene for normalization. Primer sequences can be found in Supplementary Table S3.

### RNA-sequencing and Bioinformatic Analysis

Eyes were dissected to harvest the neural retina and eye cup, which were then flash frozen using liquid nitrogen. RNA was isolated as described above. Samples were processed by Novogene Co. (Sacramento, CA, USA) using Illumina platforms. Initial bioinformatic analysis performed by Novogene included sample and data quality control, mapping to the reference genome (*Mus Musculus* GRCm39/m39), gene expression quantification, differential expression analysis, and enrichment analysis. Fragments per kilobase of transcript per million fragments mapped values were calculated based on the length and read count mapped to each gene. Principal component analysis was performed using fragments per kilobase of transcript per million fragments mapped values to compare the clustering of samples.

### Differential Expression Analysis

Differential expression analysis was performed between the PRDM13-OE + dox group and wildtype + dox group, with a cutoff of the absolute value of a log2-fold change of greater than 1 or less than −1 and an adjusted *P* value of less than 0.05. For the neural retina, of the 25,984 genes identified in our dataset, 126 genes were upregulated and 254 genes were downregulated. Genes for the visual perception volcano plot were collected from the Gene Ontology (GO) categories for *M*. *musculus* found on AmiGo2 (GO: 0007601).^21^ The retinal dystrophy genes volcano plot gene set was determined by pulling gene lists from RetNet, the Retinal Information Network for Leber congenital amaurosis, macular degeneration, and RP.^22^ Amacrine cell-related genes for the volcano plot in Figure 9H were compiled from Yan et al.^17^ After compiling the gene lists, determination of differentially expressed genes (DEGs) for each of the volcano plots were selected using the same log2-fold change and adjusted *P* value cutoffs described elsewhere in this article. Any genes not in our RNA sequencing file were removed from the curated gene lists. For the eyecup, of the 27,427 genes sequenced, 48 genes were upregulated and 51 genes were downregulated. For amacrine cell-related genes, two genes were upregulated and eight genes were downregulated.

### Enrichment Analysis

GO analysis was completed using the GO knowledge base at geneontology.org. A list of the 254 downregulated genes in the neural retina or the 51 downregulated genes of the eyecup from our differential expression analysis, described elsewhere in this article, were copied into the GO Enrichment Analysis tool, with the search parameters set to Biological Process or Cellular Component and *M*. *musculus.*^23^^–^^25^ Significant hits of interest were chosen from the list of all significant biological processes or cellular components.

### Western Blot

Retinas were collected as previously described.^20^ One retina was lysed in RIPA buffer (Thermo Fisher Scientific, Waltham, MA, USA; catalog 89900) with protease and phosphatase inhibitors (Thermo Fisher Scientific, catalog 87786). Lysis was performed using sonication, with 20 second pulses three times. Samples were run on a 4% to 12% Bis-Tris gradient gel (Thermo Fisher Scientific, NW04125BOX), then transferred to a nitrocellulose membrane using the iBlot2 dry transfer system (Thermo Fisher Scientific, IB21001). The transfer protocol was 20 V for 1 minute, 23 V for 4 minutes, and 25 V for 2 minutes. Membranes were then blocked in 5% milk in TBST for 1 hour at room temperature. Primary antibodies were diluted in 5% BSA in TBST overnight at 4°C. The following day, the membranes were washed three times in TBST, followed by a 45-minute incubation in LICOR secondary antibodies or 1-hour incubation in horseradish peroxidase secondary antibodies diluted in 5% milk in TBST. Imaging and quantification were performed using either the LI-COR Odessey CLx or GE Amersham Imager 600, depending on the secondary antibody used. At least three biological replicates per group with two technical replicates were performed for each protein of interest.

### BioRender License Information

**Figure 1.**
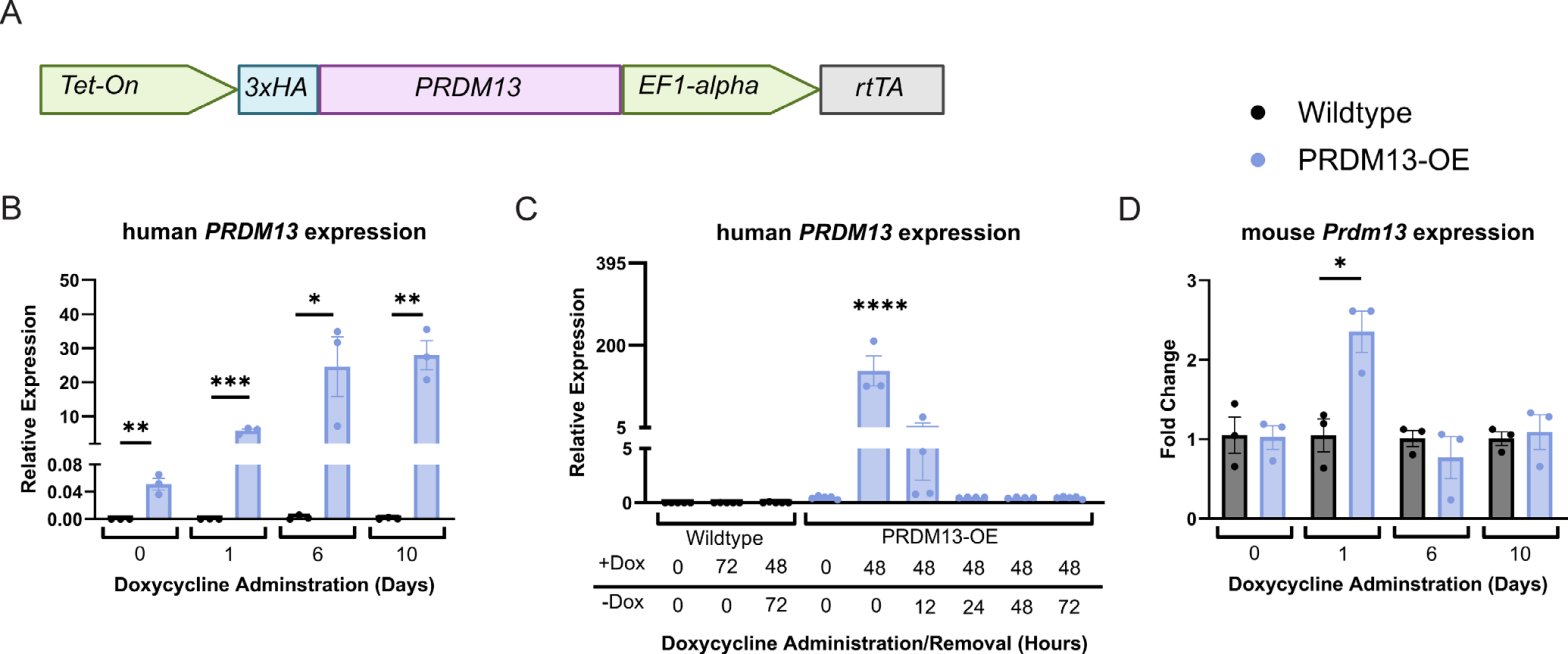
Development of a mouse model to study *PRDM13* in the retina. (**A**) Cartoon of the knock-in construct. (**B**) Human *PRDM13* relative expression in neural retina samples provided dox beginning at P28. Data was analyzed by unpaired *t* test between PRDM13-OE mice and wildtype littermates provided the same amount of dox. Error bars are SEM. (**C**) Human *PRDM13* relative expression levels from neural retinas of PRDM13-OE mice and wildtype littermates after 48 to 72 hours of dox and removal of dox for up to 72 hours. Data was analyzed by one-way ANOVA with Dunnett's multiple comparison test. Error bars are SEM. Significance is shown for all groups compared with PRDM13-OE retinas without dox. (**D**) Mouse *Prdm13* relative expression in neural retinas from PRDM13-OE mice and wildtype littermates. Data were analyzed by unpaired *t* test between PRDM13-OE mice compared with wildtype littermates provided the same amount of dox. Error bars are SD. For all tests, *N* ≥ 3 biological replicates, each representing an average of three technical replicates. * *P* < 0.05; **P < 0.01; *** *P* < 0.001; **** *P* < 0.0001.

**Figure 2.**
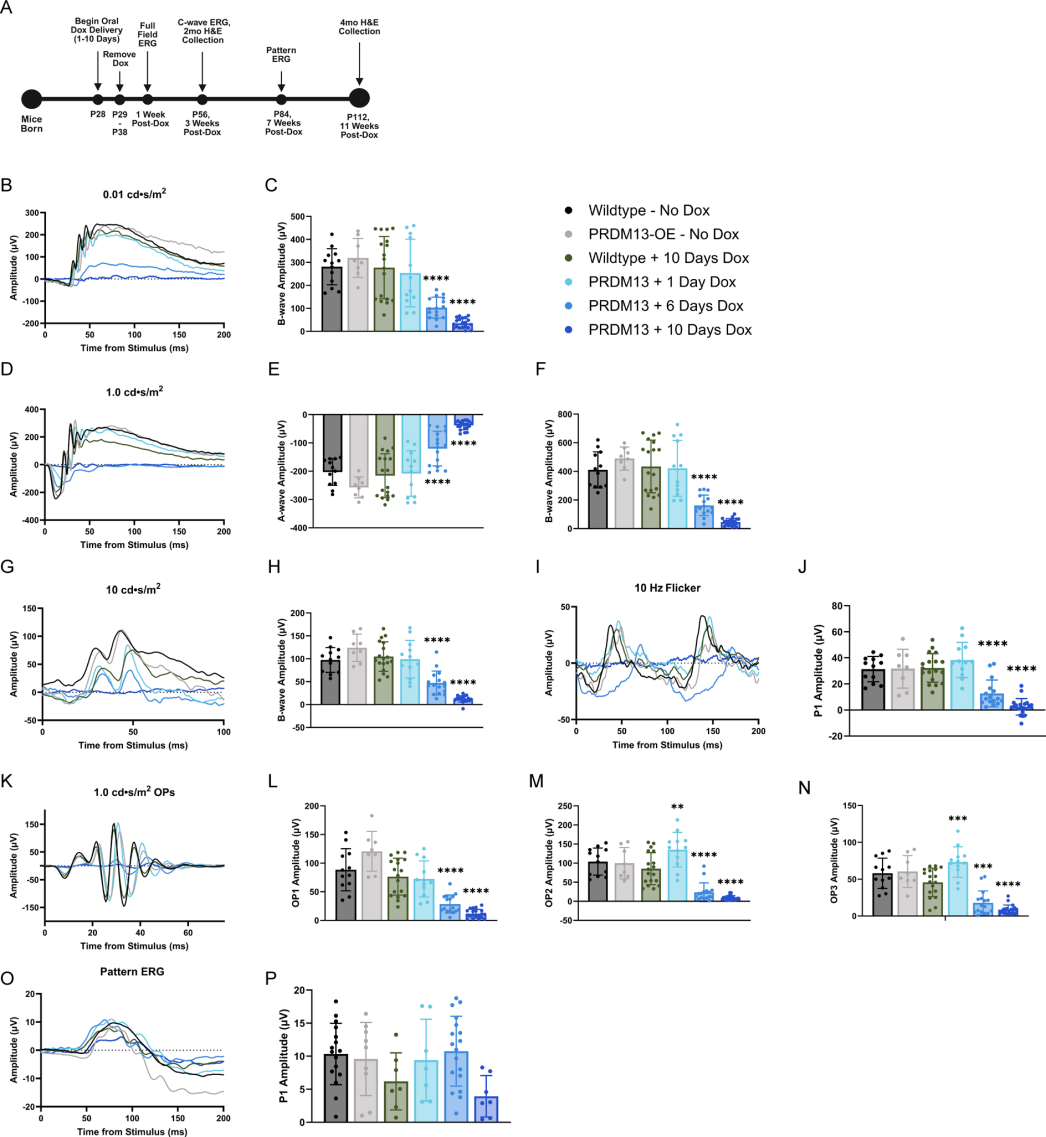
Elevated *PRDM13* for more than 6 days reduces outer retinal function. (**A**) Timeline for experimental methods. (**B**) Representative traces from individual mice and (**C**) b-wave amplitudes for scotopic ERG at the 0.01 cd•s/m^2^ light intensity setting, and (**D**) traces, (**E**) a-wave, and (**F**) b-wave amplitudes for the 1.0 cd•s/m^2^ light intensity setting. (**G**) Representative traces and (**H**) b-wave amplitudes for photopic 10.0 cd•s/m^2^ flash, and (**I**) traces and (**J**) amplitudes for the 10.0 Hz flicker. (**K**) Representative traces for the OPs and amplitudes for (**L**) OP1, (**M**) OP2, and (**N**) OP3 at the 1.0 cd•s/m^2^ light setting. (**O**) Representative traces and (**P**) amplitudes for PERG responses. Data were analyzed by one-way ANOVA with Tukey's multiple comparison's test. Significance is shown for all groups compared with wildtype with dox. Error bars are SD. *N* ≥ 8 eyes per group. ** *P* < 0.01; *** *P* < 0.001; **** *P* < 0.0001.

Some portions of figures were made using BioRender. Agreement Numbers are as follows: LM27ESBO3A (Fig. 1A); BB27ESCDPL (Fig. 2A); VO27X3GCUW (Supplementary Fig. S1B).

### Statistics

Data are reported as mean ± standard deviation unless otherwise noted in the figure legend. GraphPad Prism Software (version 10.0; La Jolla, CA, USA) was used to generate graphs and perform statistical analysis unless otherwise indicated. A *P* value of less than 0.05 was considered significant. Grubb's test was used to perform outlier analysis. Measurements were done blinded to experimental groups. Sample sizes, precise statistical testing, and post hoc methods are provided in each figure legend as well as in the Supporting Data Values file.

### Study Approval

All experiments were performed in accordance with the ARVO Statement for the Use of Animals in Ophthalmic and Visual Research, ARRIVE guidelines, and were all approved by the Animal Care and Use Committee at UT Southwestern Medical Center.

### Data Availability

Values for all data points in graphs are reported in the Supporting Data Values file. RNA sequencing raw data are available through GEO: GSE278881. All other data are provided within the main and Supplementary Files.

## Results

### Development of a Mouse Model to Study Dysregulated *PRDM13* in the Retina

Prior studies suggest a role for PRDM13 in photoreceptor maturation and maintenance, but there is a lack of knowledge for how dysregulated *PRDM13* expression affects the retina, and the regulatory targets of PRDM13 within the retina remain unknown. To investigate the impact of elevated *PRDM13* on the retina, we generated a mouse model that carries a dox-inducible human *PRDM13* gene in the *Rosa26* safe harbor locus (Fig. 1A). This allows induced expression of *PRDM13* at precisely determined times via delivery of dox, without disrupting endogenous mouse *Prdm13*, to investigate its effect on the photoreceptors and overall retinal health in a controlled manner. To test whether our human *PRDM13* was elevated within the retina after dox delivery, PRDM13-OE and wildtype littermates were provided dox beginning at P28 and continuing for 1 to 10 days. Neural retinas were collected at each timepoint, and qPCR was performed for human *PRDM13* transcripts (Fig. 1B). *PRDM13* was significantly elevated in the neural retina for all timepoints compared with wildtype littermates. We next determined how rapidly this aberrant *PRDM13* expression could be reduced with the removal of dox (Fig. 1C). PRDM13-OE mice and wildtype littermates were treated with dox for 48 to 72 hours, then dox was removed and retinas were analyzed at 12, 24, 48, or 72 hours after the removal of dox. qPCR analysis showed that 12 hours after the removal of dox, *PRDM13* expression levels are no longer significantly elevated in the retina (Fig. 1C).

### Elevated *PRDM13* Affects Its Known Regulatory Network

Unfortunately, no currently available antibodies were able to detect PRDM13 protein, even in the amacrine cell layer of wildtype mouse retinas. Because we were unable to examine PRDM13 protein directly after dox delivery, we analyzed the effects of elevated *PRDM13* on its known regulatory network. Current literature has linked *PRDM13* with genes known to be expressed during embryonic development and not in adulthood^26^; therefore, to test for functional effects of elevated PRDM13, we examined retinal tissue during embryonic development (Supplementary Fig. S1). First, we provided dox to wildtype pregnant females mated with PRDM13-OE males beginning at E13.5 of pregnancy and dissected retinas from the embryos after 24 hours. Controls were mated in the same manner, but not provided dox. qPCR for human *PRDM13* showed a significant increase in *PRDM13* within PRDM13-OE embryonic retinas that did not receive dox, indicating some leakiness of the TetOn system (Supplementary Fig. S1A). However, there was a significantly greater elevation of *PRDM13* after 24 hours of dox delivery in the PRDM13-OE embryonic retinas compared with wildtype dox-treated controls. Because *PRDM13* was significantly elevated in the embryonic retinas after 24 hours of dox delivery, we looked at key members of its regulatory network at this time via qPCR: mouse *Prdm13*, *Ptf1a*, and *Tlx3* (Supplementary Fig. S1B). Elevation of human *PRDM13* acted within its feedback loop to repress *Ptf1a*, reducing endogenous mouse *Prdm13* (Supplementary Figs. S1C, S1D). Even with the significant reduction of mouse *Prdm13* after human *PRDM13* overexpression, *Tlx3* expression was significantly reduced, indicating that the human PRDM13 protein was functioning as expected (Supplementary Fig. S1E).

Our newly developed mouse model allows for precise control of PRDM13 overexpression, acting as a tool that can be used to understand how PRDM13 dysregulation within the retina impacts cell development, maintenance, and survival. To initially bypass the confounding effect of the feedback regulatory loop during development, where mouse *Prdm13* was significantly reduced, we tested whether dox delivery in the adult retina could be used to decipher the impact of elevated *PRDM13* and its retinal regulatory targets, which can later be examined during development. To assess whether elevated human *PRDM13* also significantly altered endogenous mouse *Prdm13* in the adult retina, we performed qPCR on retinas collected after 1, 6, or 10 days of dox delivered beginning at P28 (Fig. 1D). We discovered that although 1 day of dox caused a significant increase in mouse *Prdm13*, this was reduced to wildtype expression after 6 and 10 days of dox, although human *PRDM13* remained significantly elevated throughout the 10 days (Figs. 1B, 1D).

### Elevated *PRDM13* Reduces Outer Retina Visual Function

To decipher which retinal cell types may be affected by dysregulated *PRDM13*, we elevated *PRDM13* in the adult retina beginning at P28, using 1, 6, or 10 days of dox delivery. We tested the mice for changes in visual function by scotopic and photopic ERG 1 week after the removal of dox, c-wave ERG at 3 weeks after the removal of dox, PERG at 7 weeks after the removal of dox, and hematoxylin and eosin (H&E) staining performed on retinal sections at both 2 and 4 months of age to assess retinal morphology (Fig. 2A). Tests were performed at these different ages to allow mice to be placed under anesthesia, but have time for recovery between testing methods, following our institutional guidelines.

Wildtype mice provided with dox for 1 to 10 days showed no significant loss of either scotopic or photopic ERG amplitudes compared with wildtype mice without dox for all light intensities analyzed (Supplementary Fig. S2). Therefore, we compared PRDM13-OE mice that were provided with dox for 1 to 10 days with wildtype mice provided dox for the full 10 days. PRDM13-OE and wildtype littermates without dox were also analyzed. 0.01 cd·s/m^2^ scotopic ERG, representative of rod photoreceptor function, showed a significant decrease in b-wave amplitudes for the PRDM13-OE mice provided with dox for more than 6 days compared with the dox-treated wildtype controls (Fig. 2B, 2C). We found that 1.0 cd·s/m^2^ scotopic ERG, representative of global retinal function, showed a significant loss of a-wave (photoreceptor) and b-wave (bipolar) amplitudes in PRDM13-OE mice that received dox for more than 6 days compared with the dox-treated wildtype controls (Fig. 2D–F). Photopic ERG results, representing cone photoreceptor function, mimic those of the scotopic settings, demonstrating significantly decreased amplitudes for PRDM13-OE mice with more than 6 days of dox compared with dox-treated wildtype controls at both the 10 cd·s/m^2^ flash (Figs. 2G, 2H) and 10 Hz flicker settings (Figs. 2I, 2J). Because PRDM13 has been associated with amacrine cell subtype fate choice and survival,^14^ we analyzed the oscillatory potentials (OPs) 1, 2, and 3 in the 1.0 cd·s/m^2^ scotopic ERGs (Figs. 2K–N). Interestingly, after 1 day of dox, the PRDM13-OE mice showed a significant elevation of OP2 and OP3 compared with dox-treated controls, but a significant reduction in all three OPs after 6 days of dox.

We next examined retinal cells that have not previously been associated with PRDM13. At 3 weeks after the removal of dox, mice underwent c-wave ERG to assess the functional interaction between the RPE and the photoreceptor outer segments (Supplementary Fig. S3). Similar to scotopic and photopic ERGs, c-wave ERG amplitudes were significantly reduced in PRDM13-OE mice that received dox for more than 6 days compared with wildtype controls that received dox for the same duration. However, we did note an unexpected reduction in c-wave amplitudes in wildtype mice with 1 and 10 days of dox, indicating an adverse effect of dox alone on this functional testing method. At 7 weeks after the removal of dox, mice underwent PERG to test for retinal ganglion cell function (Figs. 2O, 2P). No significant difference was noted for the PRDM13-OE mice compared with controls when treated with dox for up to 10 days.

### Elevated *PRDM13* Causes Retinal Degeneration But Stabilizes When *PRDM13* Expression Is No Longer Dysregulated

Because elevation of *PRDM13* caused a loss of visual function consistent with a retinal degenerative phenotype, we imaged individual mice using OCT throughout the 10 days of dox delivery and over 8 weeks after the removal of dox to assess retinal morphology (Supplementary Fig. S4). Wildtype mice displayed no change in TRT from baseline through 10 days of dox delivery (Supplementary Fig. S4B), as well as between baseline and through 8 weeks after removal of dox (Supplementary Fig. S4C). Interestingly, the PRDM13-OE mice showed no significant difference in TRT until 10 days of dox delivery, where the TRT was significantly reduced compared with the PRDM13-OE baseline retinal thickness (Supplementary Fig. S4D). TRT continued to decrease 1 week after the removal of dox, but then stabilized and remained a similar thickness through 8 weeks after the removal of dox, indicating a halt in the progression of retinal degeneration (Supplementary Fig. S4E).

### Visual Function Rebounds After *PRDM13* Is No Longer Dysregulated

**Figure 3.**
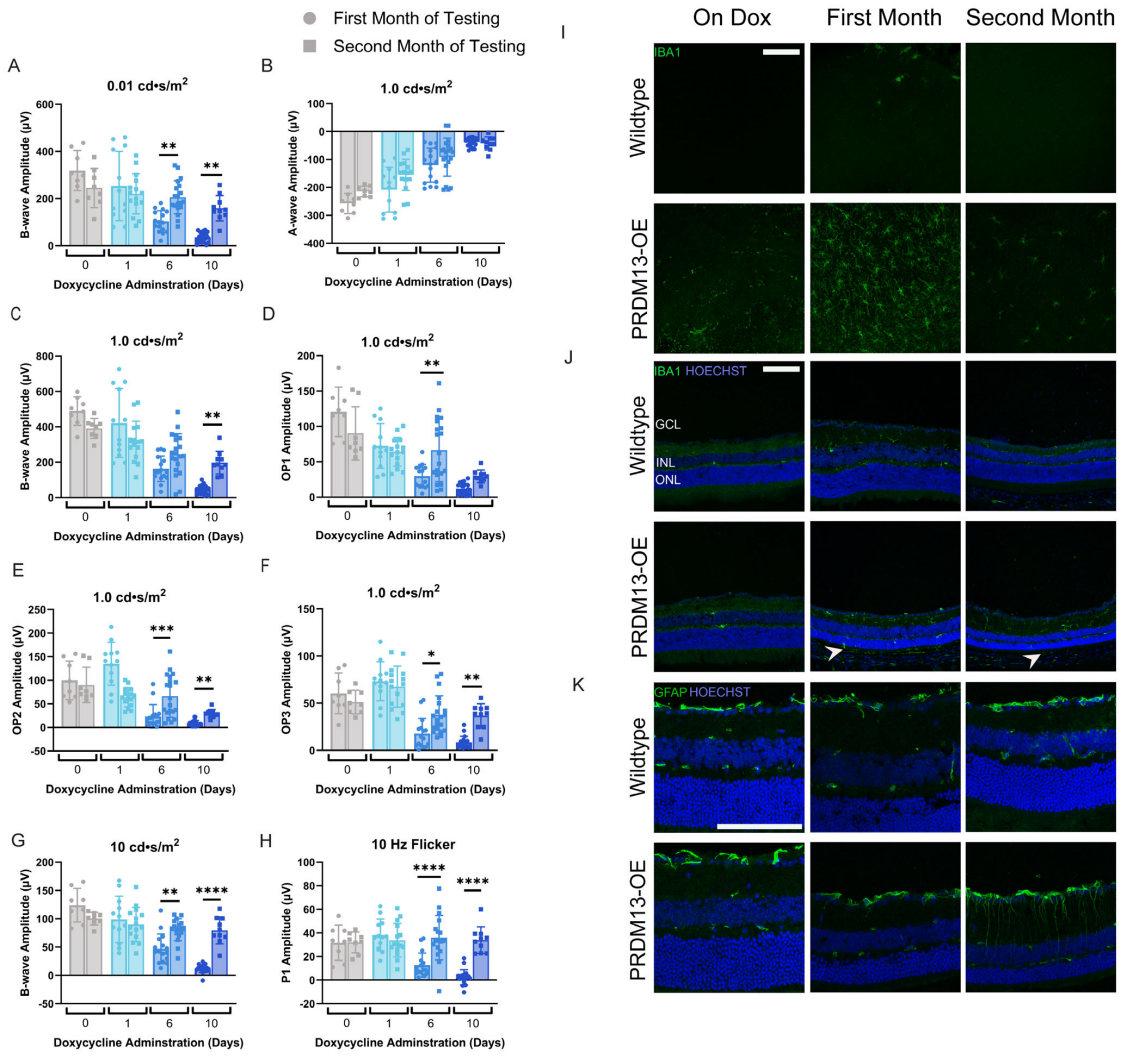
Visual function rebounds after *PRDM13* expression is no longer elevated. PRDM13-OE mice provided dox for up to 10 days underwent scotopic and photopic ERG 1 and 3 weeks after removal of dox (first and second month of testing, respectively). ERG amplitudes were analyzed for the (**A**) 0.01 cd•s/m^2^ b-wave, (**B**) 1.0 cd•s/m^2^ a-wave, (**C**) 1.0 cd•s/m^2^ b-wave, (**D**) 1.0 cd•s/m^2^ OP1, (**E**) 1.0 cd•s/m^2^ OP2, (**F**) 1.0 cd•s/m^2^ OP3, (**G**) light-adapted 10 cd•s/m^2^ b-wave, and (**H**) 10 Hz flicker. Data were analyzed by two-way ANOVA with Šídák's multiple comparison test (scotopic ERGs) or Tukey's multiple comparison's test (photopic ERGs). Error bars are SD. Significance is shown between the same group for each imaging date. * *P* < 0.05; ** *P* < 0.01; *** *P* < 0.001; **** *P* < 0.0001. *N* ≥ 8 eyes. (**I**) Retinal flat mounts and (**J, K**) sagittal sections for wildtype and PRDM13-OE mice provided dox for 3 days (on dox group) or 10 days of dox followed by 1 and 3 weeks after removal of dox (first and second month of testing, respectively) were stained for either (**I, J**) IBA1 or (**K**) glial fibrillary acidic protein. Retinal flat mounts shown in (**I**) are from peripheral retina. *White arrowheads* in (**J**) mark the presence of IBA1 staining within the subretinal space. Hoechst, nuclei. GCL, ganglion cell layer. Three biological replicates with at least two technical replicates each were analyzed. *Scale* *bar*, 100 µm.

Elevated *PRDM13* caused a significant decline in visual function, and OCT indicated that the removal of dox halted the decline in TRT; thus, we tested visual function between 1 and 3 weeks after dox removal to see whether there was a progressive loss of function after *PRDM13* was no longer dysregulated (Fig. 3). PRDM13-OE mice treated for longer durations of dox (6 and 10 days) showed a significant elevation in visual function for all but the 1.0 cd·s/m^2^ scotopic ERG a-wave amplitudes between 1 and 3 weeks after dox removal (Fig. 3).

### Activated Immune Responses Persist for at Least 1 Week After PRDM13 Is No Longer Elevated

We hypothesized that this delayed restoration of function is due to the retinal cells remaining stressed for a prolonged window of time after the dysregulation of *PRDM13*. To test this, PRDM13-OE and wildtype mice were treated with dox for 3 days (on dox group), 10 days with 1 week of recovery (reflecting the first month), and 10 days with 3 weeks of recovery (reflecting the second month). IBA1 immunostaining performed on retinal flat mounts showed a mild increase in IBA1+ microglia in PRDM13-OE retinas compared with wildtype retinas while on dox, with a striking elevation of microglia in the first month that was lessened by the second month (Fig. 3I). To test whether the microglia responded to stress from the photoreceptors, IBA1 immunostaining was performed on sagittal sections of the retina (Fig. 3J). No microglia were detected in the subretinal space in wildtype mice. In contrast, microglia migrated into the subretinal space of the PRDM13-OE mice in the first month (1 week after removal of dox; *white arrowhead*), with reduced numbers of microglia present in the subretinal space by the second month (*white arrowhead*). Along with activation and migration of the microglia, Müller glia activation was analyzed by staining with glial fibrillary acidic protein (Fig. 3K). Wildtype mice showed no glial activation, but PRDM13-OE mice showed mild glial activation in the first month, with striking activation by the second month. No glial activation was detected in the PRDM13-OE retinas three months after the removal of dox (Supplementary Fig. S5).

### Elevated PRDM13 Causes a Nonprogressive Reduction in the ONL

**Figure 4.**
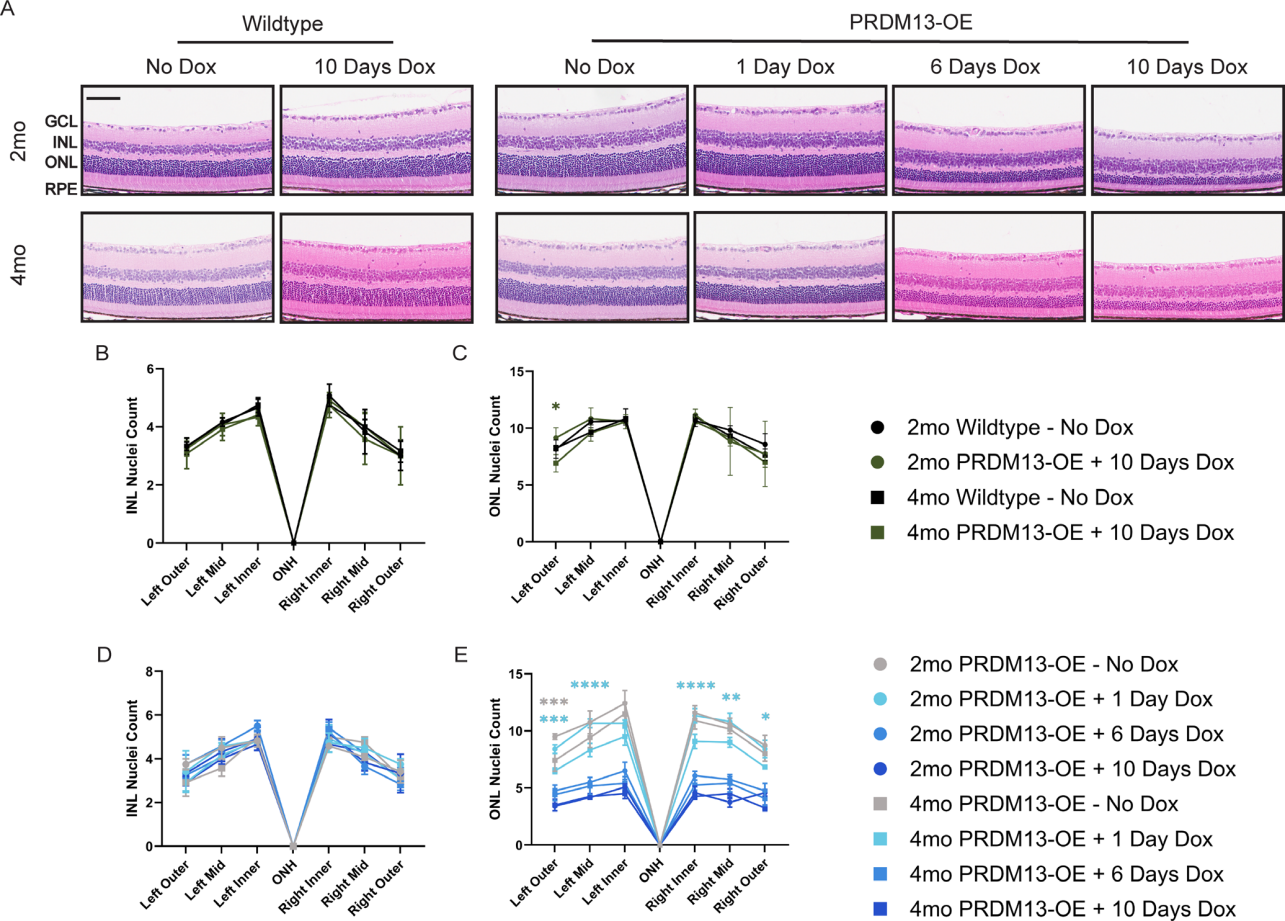
Elevated *PRDM13* causes photoreceptor cell death. (**A**) Representative H&E–stained retinal sections for PRDM13-OE and wildtype littermates after the removal of dox (2 and 4 months of age), after providing dox for 1, 6, or 10 days at P28. *Scale bar*, 50 µm. GCL, ganglion cell layer. Nuclei counts for the (**B**) INL and (**C**) ONL of untreated and dox-treated wildtype mice at 2 and 4 months of age. Nuclei counts for the (**D**) INL and (**E**) ONL of untreated and dox-treated PRDM13-OE mice at 2 and 4 months of age. Data were analyzed by two-way ANOVA with Tukey's multiple comparison's test. Error bars are SD. Significance is shown between the untreated or dox-treated mice between 2 and 4 months of age and asterisks are colored to match the experimental group they represent. *N* = 3 mice per group. * *P* < 0.05; ** *P* < 0.01; *** *P* < 0.001; **** *P* < 0.0001.

We hypothesized that the nonrecovered loss of vision after *PRDM13* overexpression was due to the loss of the photoreceptors in the ONL. To test this hypothesis, we quantified the retinal cell layers on H&E sections from mice at 3 weeks after the removal of dox (2 months of age) and 11 weeks after the removal of dox (4 months of age) (Figs. 4A–E). We examined the thickness of the INL—where the amacrine, horizontal, and bipolar cells reside—and the ONL at all regions of the retina spanning from the optic nerve head and compared the thickness between the same groups between 2 and 4 months of age (Fig. 4B–E). In wildtype mice without dox, no changes in INL or ONL thickness were noted (Fig. 4B, 4C). After 10 days of dox, no difference was detected in the INL, but one peripheral region showed a significant decrease in the ONL (Figs. 4B, 4C). Comparable with wildtype littermates, the PRDM13-OE mice with and without dox for up to 10 days showed no difference in the INL (Fig. 4D). The PRDM13-OE mice without dox treatment also showed one peripheral region of the ONL to be significantly reduced (Fig. 4E). However, 1 day of dox delivery to the PRDM13-OE mice at P28 caused a significant reduction in the ONL, suggesting a progression of degeneration with a short duration of dox in these mice (Fig. 4E). PRDM13-OE mice treated with dox for 6 or 10 days at P28 showed a significantly reduced ONL compared with baseline, but no further degeneration occurred between 2 and 4 months of age (Fig. 4E).

### Elevated PRDM13 Does Not Cause Large-scale Adverse Effects to Retinal Cell Morphology, Apart From Photoreceptors

**Figure 5.**
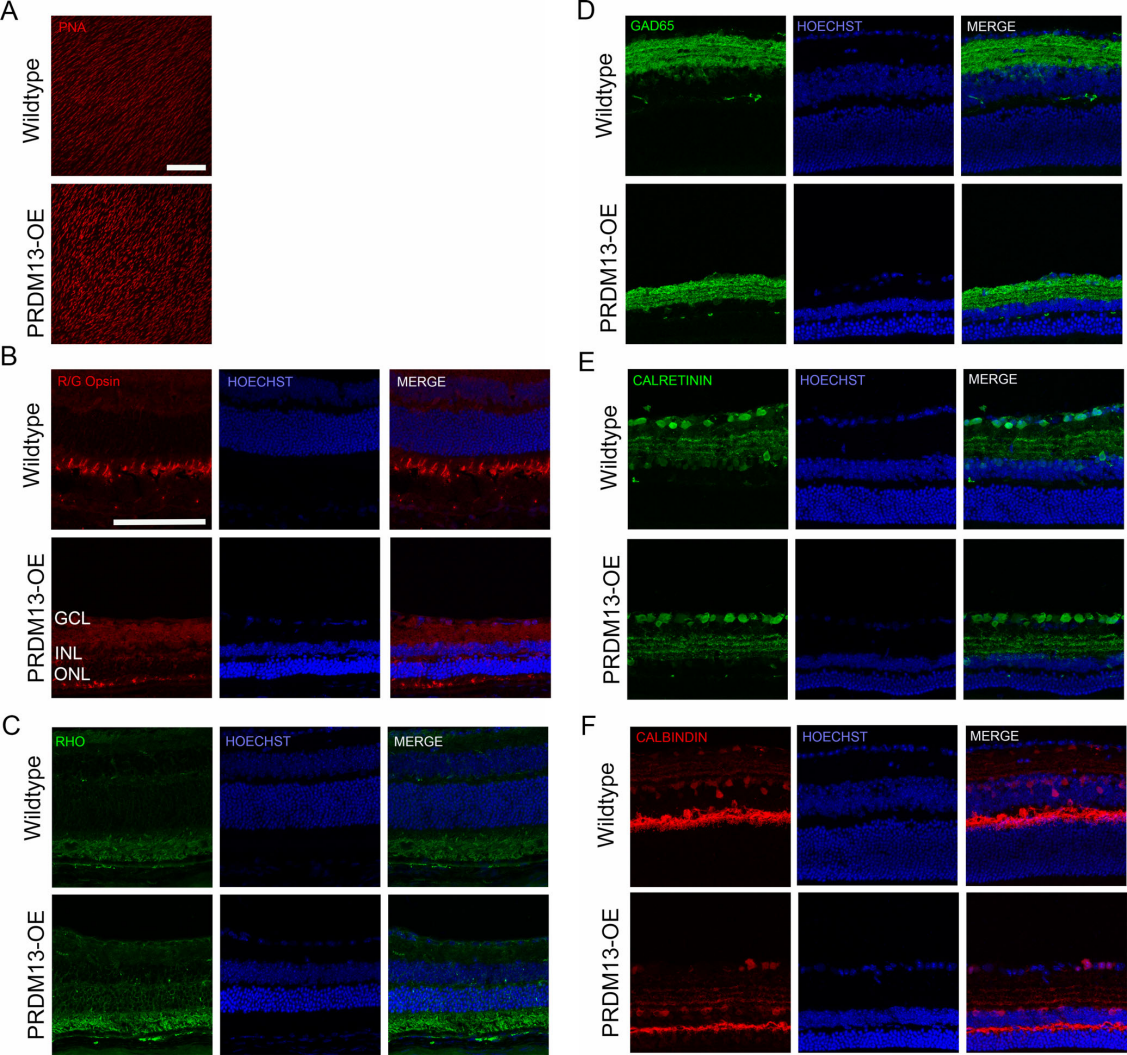
Elevated *PRDM13* causes a loss of rod photoreceptors and collapsed cone photoreceptor morphology. Representative (**A**) retinal flat mounts or (**B–F**) sagittal sections for PRDM13-OE mice and wildtype littermates at P56 with dox provided at P28 for 10 days. Retinas were stained for (**A**) peanut agglutinin, (**B**) R/G opsin, (**C**) rhodopsin, (**D**) GAD65, (**E**) calretinin, and (**F**) calbindin. Hoechst, nuclei. GCL, ganglion cell layer. Retinal flat mounts shown in (**B**) are from the peripheral retina. Three biological replicates with at least one technical replicate each were analyzed. *Scale* *bar*, 100 µm.

We found that there was a significant loss of the ONL, and not the INL, after *PRDM13* elevation in the adult retina. Thus, we examined whether the major cell types of the neural retina were morphologically abnormal. PRDM13-OE and wildtype littermates were provided dox for 10 days, and eyes underwent immunostaining for key retinal cell type markers after the time in which retinal degeneration no longer progresses, either 3 weeks after the removal of dox (Fig. 5) or 11 weeks after the removal of dox (Supplementary Fig. S6). Retinal flat mounts stained for peanut agglutinin showed no major differences in the overall cone number between PRDM13-OE mice and wildtype controls (Fig. 5A). However, R/G opsin, reflecting cone photoreceptors, showed a collapsed morphology indicative of the loss of the rods in the PRDM13-OE retinas compared with controls (Fig. 5B and Supplementary Fig. S6A). RHO staining confirmed the loss of the rod photoreceptors in the PRDM13-OE retinas compared with controls (Figs. 5C, Supplementary S6B). Because PRDM13 is known to be expressed specifically in the amacrine cells during adulthood, we looked at multiple amacrine cell markers; GAD65 (Fig. 5D), calretinin (Fig. 5E), and calbindin (Fig. 5F). We found no major morphological changes between PRDM13-OE and wildtype littermates. Additionally, no major morphological changes were noted for the retinal ganglion cells (Supplementary Fig. S6C) or the bipolar cells (Supplementary Fig. S6D).

### Elevated *PRDM13* Alters the Transcriptomic Profile of the Neural Retina

**Figure 6.**
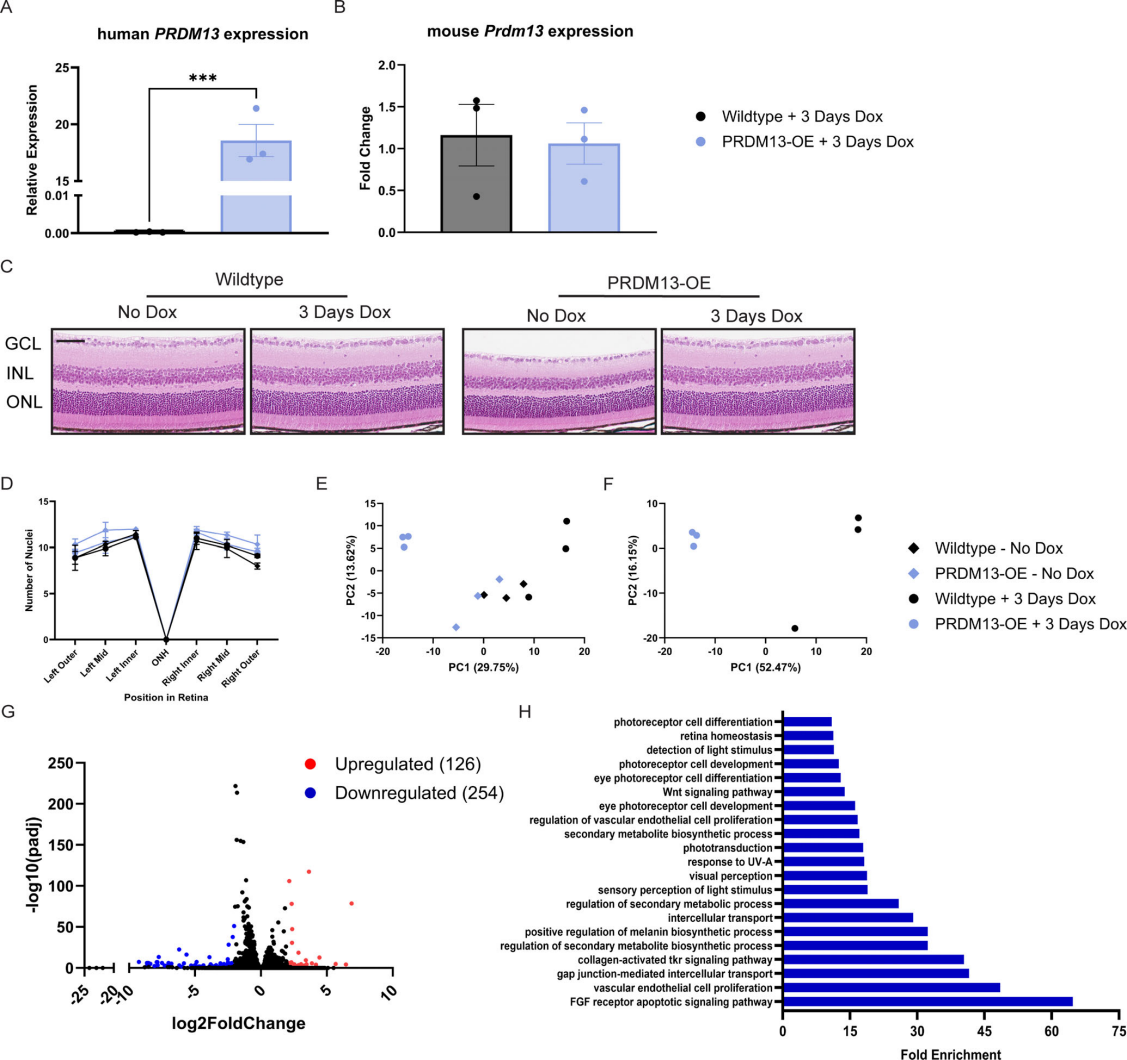
Elevated *PRDM13* alters transcriptomic profiles of the neural retina. PRDM13-OE mice and wildtype littermates were untreated or treated with dox for 3 days beginning at P28. Retinas underwent qPCR for (**A**) human *PRDM13* and (**B**) mouse *Prdm13* expression levels. Data were analyzed by unpaired *t* test. Error bars are SD. *N* = 3 biological replicates, each representing an average of three technical replicates**.** Retinal sections were obtained and (**C**) stained with H&E. *Scale* *bar*, 50 µm. GCL, ganglion cell layer. (**D**) Quantification of the ONL was performed and analyzed by two-way ANOVA with Tukey's multiple comparison's test. Error bars are SD. *N* = 3 eyes per group. Using the same groups, neural retinas were collected, RNA was isolated, and RNA-sequencing was performed. (**E**) Principal component analysis plot for all biological replicates and control groups. (**F**) Principal component analysis plot for PRDM13-OE and wildtype littermates treated with dox. (**G**) PRDM13-OE and wildtype retinas that both received dox were compared for differential gene expressions. Upregulated (*red*, 126 genes) and downregulated (*blue*, 254 genes) genes were determined using a log2-fold change of greater than 1 or less than −1 and an adjusted *P* value of less than 0.05. (**H**) GO analysis of the downregulated genes.

Although elevated *PRDM13* did not cause a detectable morphological abnormality in the retinal cell types outside of the photoreceptors, it may cause smaller but significant changes in more precise subtypes of cells. This finding is supported by the fact that loss of *Prdm13* depleted subpopulations of amacrine cells, but not all amacrine cells.^14^ To investigate this, we used RNA sequencing on neural retinas and eye cups (containing the RPE, choroid, and sclera) from PRDM13-OE mice and wildtype controls with and without delivery of dox at P28. Previously, we found that dox delivery for 24 hours caused a significant elevation in mouse *Prdm13*, but that gene expression was no longer elevated with longer durations of dox (Fig. 1D). To choose a dox duration that reduced the risk of a confounding effect from reduction of endogenous *Prdm13*, we tested retinal samples via qPCR and found that human *PRDM13* was significantly elevated after 3 days of dox, but there was no change in expression of mouse *Prdm13* (Figs. 6A, 6B). We examined H&E retinal sections from the mice that received dox for 3 days, as well as mice not provided with dox, and found that there was no detectable change in ONL thickness with 3 days of dox delivery (Figs. 6C, 6D). Thus, dox delivery for 3 days significantly elevates human *PRDM13* without affecting the endogenous mouse *Prdm13* and is before photoreceptor cell death. Therefore, we provided dox for 3 days and then neural retina and eye cups were collected and analyzed by RNA sequencing.

Principal component analysis of the neural retina samples showed that PRDM13-OE and wildtype mice that were not provided dox clustered similarly, whereas wildtype mice provided with dox and PRDM13-OE mice provided with dox clustered separately (Fig. 6E). To account for any confounding effect of dox, PRDM13-OE mice which received dox and wildtype mice that received dox were used for further analysis (Fig. 6F). DEGs between PRDM13-OE and wildtype littermates were determined using cutoffs of a log2 fold change of less than −1 or greater than 1 and an adjusted *P* value of less than 0.05 (Fig. 6G). In the neural retina samples, we found 126 genes were significantly upregulated and 254 genes significantly downregulated. We subjected these DEGs to GO analysis and found that no biological processes or cellular components were significant for the 126 upregulated DEGs. Supporting the role of PRDM13 as a transcriptional repressor, GO analysis of the 254 downregulated DEGs showed genes associated with biological processes including retina homeostasis, photoreceptor and retinal development, WNT and FGF signaling, and response to light stimulus (Fig. 6H).

Principal component analysis (Supplementary Figs. S7A, S7B) and DEG analysis for the eye cup samples were performed following the same log2-fold and *P* value cutoffs as the neural retina samples. We identified 48 significantly upregulated and 51 significantly downregulated genes between the PRDM13-OE and wildtype eye cups (Supplementary Fig. S7C). Similar to the neural retina samples, upregulated DEGs in the eye cup samples from PRDM13-OE mice compared with wildtype littermates underwent GO analysis, and no biological processes or cellular components were significant. However, GO analysis of downregulated DEGs returned significant biological processes and cellular components (Supplementary Figs. S7D, S7E). All significant GO terms for the eye cup samples related to the photoreceptors and visual perception.

**Figure 7.**
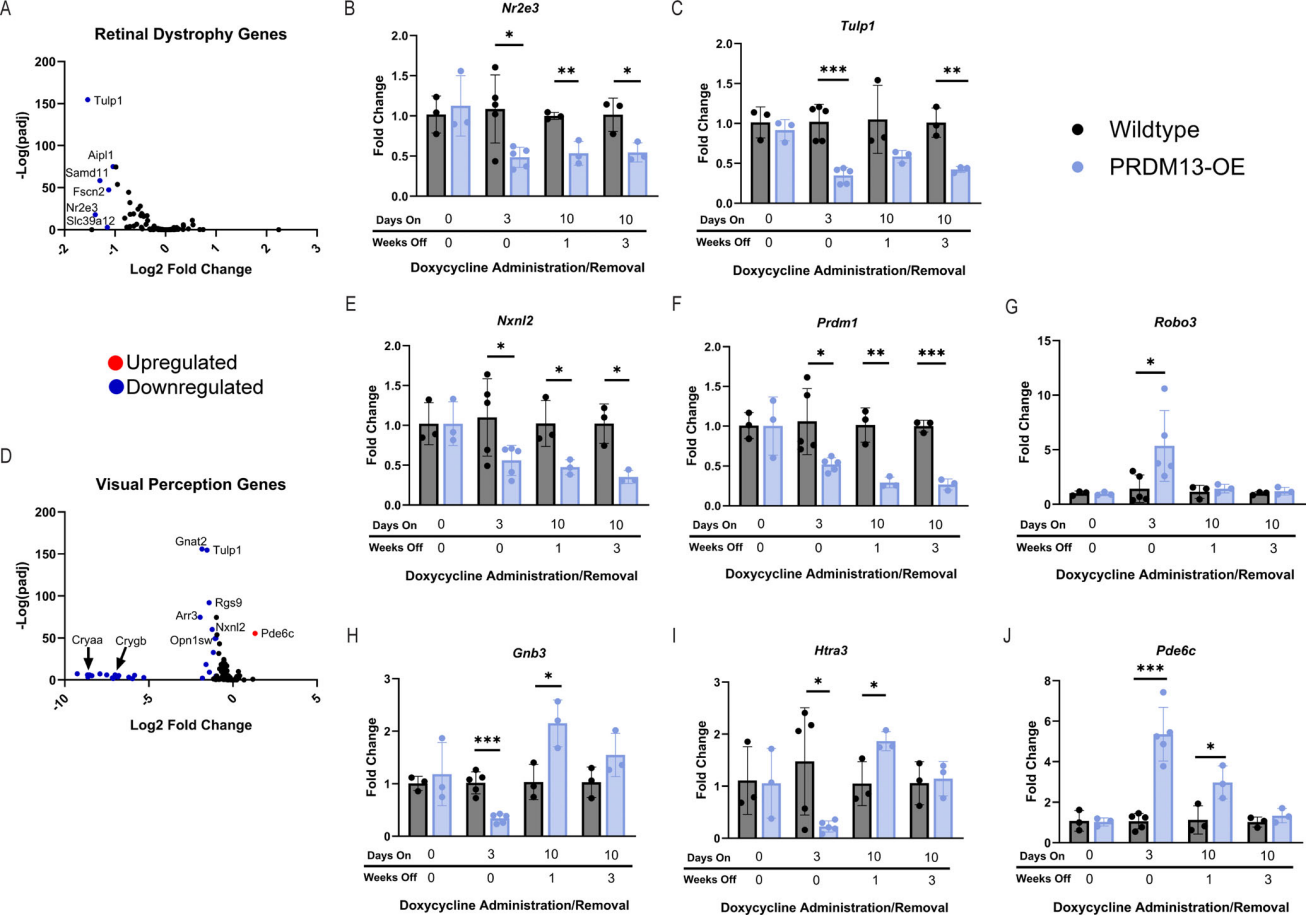
Exogenous *PRDM13* expression is associated with altered expression of known retinal dystrophy and visual perception genes. Differential gene expression for (**A**) genes known to be involved in retinal dystrophies with qPCR performed for neural retinas from PRDM13-OE mice and wildtype littermates without dox, after 3 days of dox, and 1 or 3 weeks after removal of 10 days of dox for (**B**) *Nr2e3* and (**C**) *Tulp1.* Differential gene expression of (**D**) genes involved in visual perception and qPCR for (**E**) *Nxnl2*, (**F**) *Prdm1*, (**G**) *Robo3*, (**H**) *Gnb3*, (**I**) *Htr3a*, and (**J**) *Pde6c*. Analysis was performed using unpaired *t* tests between wildtype and PRDM13-OE retinas which received the same amount of time on and off dox. Error bars are SD. ** *P* < 0.01; *** *P* < 0.001. *N* ≥ 3 biological replicates each representing an average of three technical replicates.

To further investigate the DEGs found within the neural retina samples, we compiled a list of genes associated with Leber congenital amaurosis, macular degeneration and RP^22^ and found that genes linked to these diseases were repressed in the PRDM13-OE retinas compared with controls (Fig. 7A). These included *Nr2e3* (Fig. 7B) and *Tulp1* (Fig. 7C). In addition, critical genes linked to visual perception were differentially expressed in our PRDM13-OE retinas compared with controls (Fig. 7D).

We then validated a subset of these genes of interest using qPCR (Figs. 7B, 7C, 7E–J). Neural retina tissue was collected, and RNA was isolated from mice that received no dox, 3 days of dox, 10 days of dox followed by 1 week of recovery (the same time point as our initial ERG analysis in Fig. 2), and 10 days of dox followed by 3 weeks of recovery (our follow-up ERG analysis in Fig. 3). We examined genes involved in retinal development (*Robo3*, *Htra3*, *Tulp1*, and *Prdm1*), photoreceptor maintenance (*Nxnl2* and *Nr2e3*), and phototransduction (*Pde6c* and *Gnb3*). We hypothesized that these genes would be altered significantly while *PRDM13* was overexpressed, but that they would be set in their new normal state by 1 week after the removal of dox. Interestingly, we found that some—*Prdm1*, *Nr2e3*, *Tulp1*, *Nxnl2*, and *Robo3*—but not all, genes followed our hypothesis. Our analysis of *Htra3*, *Pde6c*, and *Gnb3* showed that there was still transcriptional dysregulation 1 week after the removal of dox, that then returned to wildtype expression levels 3 weeks after dox removal. Of note, this corresponds to the time in which glial activation persists following PRDM13 elevation (Figs. 3I–K).

**Figure 8.**
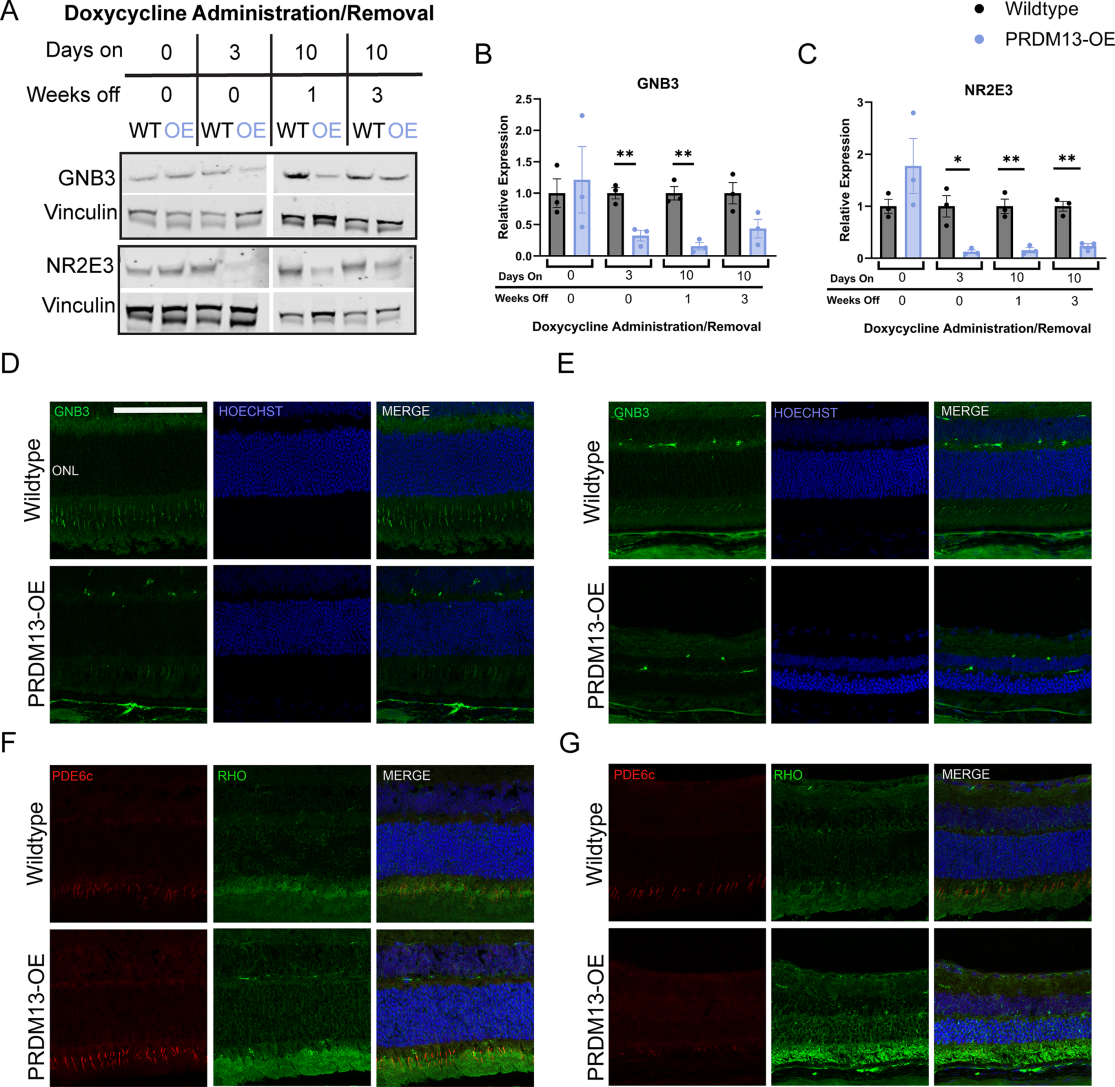
Protein expression mimics changes seen at the transcriptional level after elevated *PRDM13*. Retinas from wildtype and PRDM13-OE littermates were collected from the following groups: untreated, treated with dox for 3 days and collected on the third day, treated with dox for 10 days and collected 1 or 3 weeks after removal of dox. Western blot was performed for (**A, B**) GNB3 and (**A, C**) NR2E3. Vinculin was used as a loading control. Statistics shown are for unpaired *t* test between the wildtype and PRDM13-OE groups, which received the same amount of time on and off dox. Error bars are SD. *N* = 3 biological replicates, which represent an average of two technical replicates. * *P* < 0.05; ** *P* < 0.01; *** *P* < 0.001. Retinal sections from PRDM13-OE and wildtype littermates provided with dox for (**D, F**) 3 days or (**E, G**) 3 weeks after removal of a 10-day dox treatment were stained for (**D, E**) GNB3 (*green*) or (**F, G**) rhodopsin (*green*) and PDE6C (*red*). Three biological replicates with at least two technical replicates each analyzed. *Scale* *bar*, 100 µm.

We further validated select genes of interest at the protein level to determine how these transcriptional changes affect protein expression. We found that the GNB3 protein is significantly reduced when *PRDM13* is elevated and remains low 1 week after dox removal (Figs. 8A, 8B). It appears to be increasing toward wildtype levels by 3 weeks after the removal of dox (Figs. 8A, 8B). This sustained decrease in GNB3 was also evident via immunostaining of retinal sections from PRDM13-OE and wildtype mice provided 3 days of dox (Fig. 8D), as well as 3 weeks after 10 days of dox delivery (Fig. 8E). NR2E3 follows a similar pattern, where the protein is decreased significantly in PRDM13-OE mice with elevation of *PRDM13* and remains lowly expressed 3 weeks after 10 days of dox delivery (Figs. 8A, 8C).

Prior studies have shown that the loss of NR2E3 in the rd7 mouse model leads to the development of hybrid rod photoreceptors that express some cone photoreceptor proteins, including PDE6c.^27^^,^^28^ Because our RNA sequencing data showed both a loss of *Nr2e3* expression (Fig. 7B) and an increase in *Pde6c* expression (Fig. 7J), we performed immunostaining for PDE6C and rhodopsin on retinal sections from wildtype and PRDM13-OE mice that received dox for 3 days or 3 weeks after 10 days of dox delivery (Figs. 8F, 8G). We found that PDE6C fluorescence intensity was increased in PRDM13-OE mouse retinas compared with wildtype retinas after 3 days of dox, but did not appear to be expressed within rod photoreceptors marked by rhodopsin (Fig. 8F). Three weeks following 10 days of dox delivery, PDE6C was no longer increased in the PRDM13-OE retinas compared with wildtype retinas, and still did not appear to be expressed in the rod photoreceptors (Fig. 8G).

**Figure 9.**
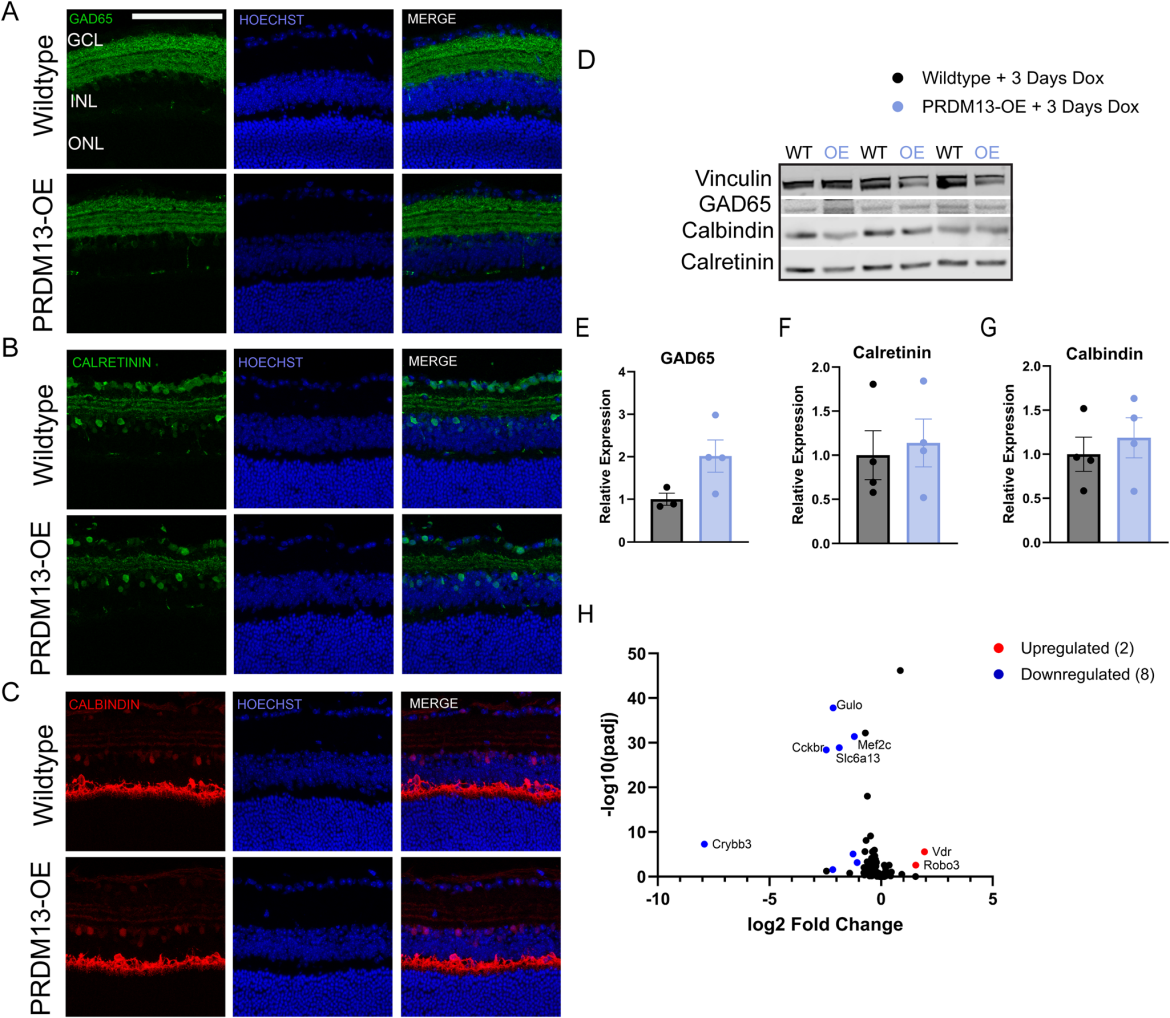
Elevated *PRDM13* alters expression of markers for amacrine cell subpopulations. Retinal sections from PRDM13-OE and wildtype mice provided dox for 3 days were stained for (**A**) GAD65, (**B**) calretinin, and (**C**) calbindin. Hoechst, nuclei. GCL, ganglion cell layer. Three biological replicates with three technical replicates each were analyzed. *Scale* *bar*, 100 µm. Neural retinas from PRDM13-OE and wildtype littermates provided 3 days of dox underwent Western blot for (**D, E**) GAD65, (**D, F**) calretinin, and (**D, G**) calbindin. Vinculin was used as a loading control. Statistics shown are for unpaired *t* test. Error bars are SD. *N* ≥ 3 biological replicates which represent an average of two technical replicates. (**H**) Differential gene expression for known amacrine cell markers show a significant upregulation of two genes and downregulation of eight genes.

**Figure 10.**
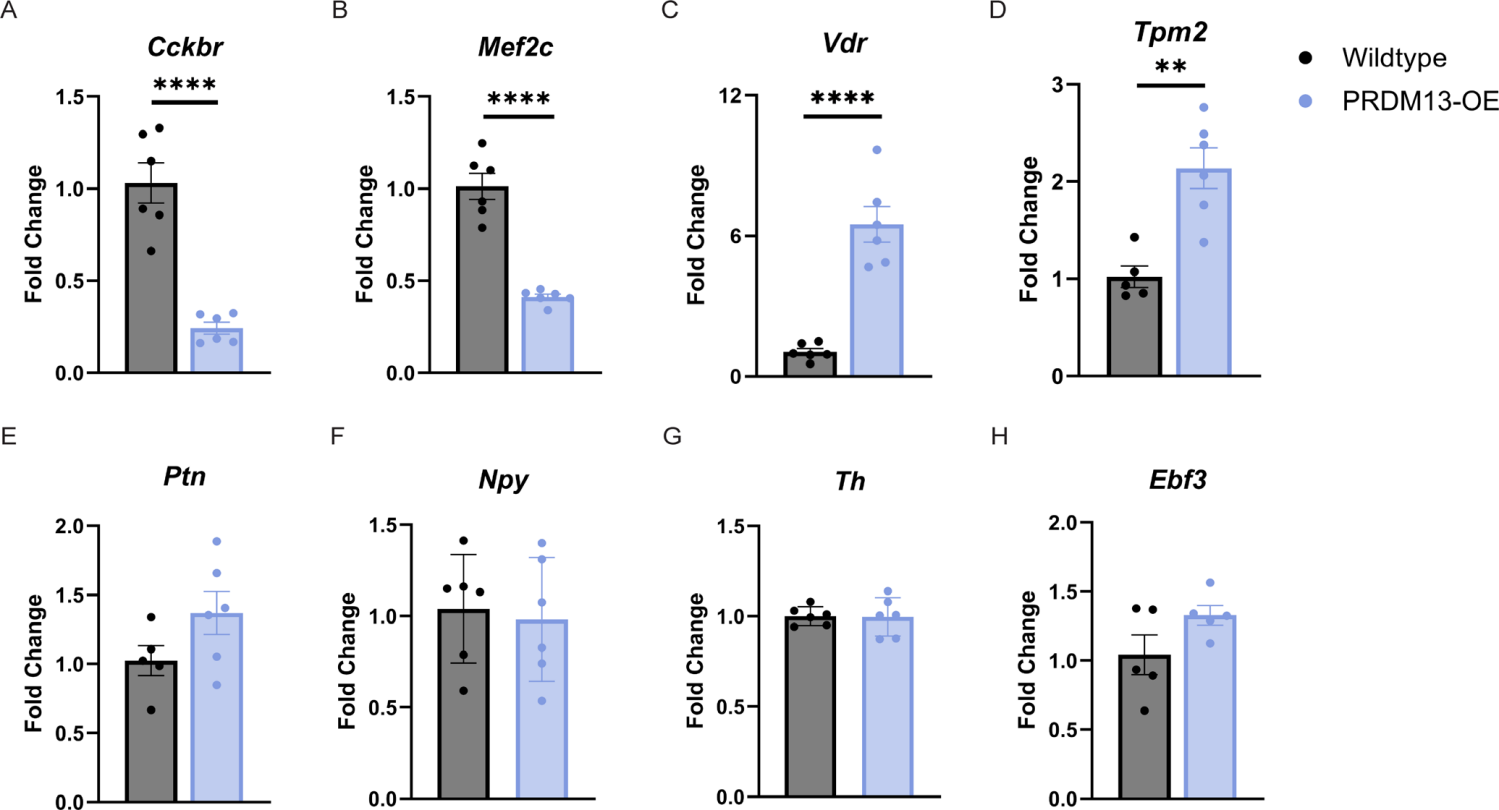
Elevated *PRDM13* alters transcription of amacrine cell-related genes. PRDM13-OE and wildtype littermates were provided dox for 3 days beginning at P28. Retinas were analyzed by qPCR for (**A**) *Cckbr*, (**B**) *Mef2c*, (**C**) *Vdr*, (**D**) *Tpm2*, (**E**) *Ptn*, (**F**) *Npy*, (**G**) *Th*, and (**H**) *Ebf3*. Data were analyzed by unpaired *t* test. Error bars are SD. ** *P* < 0.01, **** *P* < 0.0001. *N* ≥ 5 biological replicates, each an average of three technical replicates.

Although we did not see any major morphological changes to the amacrine cells after 10 days of elevated *PRDM13* (Figs. 5D–F), we tested whether there were any transient effects of dysregulated *PRDM13* on amacrine cell markers. We stained retinal sections from wildtype and PRDM13-OE mice provided dox for 3 days for GAD65, calretinin, and calbindin (Figs. 9A–C). We also performed western blotting to quantify protein levels (Figs. 9D–G). We found no significant changes in these proteins between the PRDM13-OE and wildtype retinas; however, GAD65 showed a nonsignificant but trending increase in protein expression after dysregulation of *PRDM13*. To further investigate amacrine subpopulations, we compiled a list of 139 amacrine cell-related genes and assessed their expression in our neural retina RNA sequencing dataset.^14^^,^^17^ Using DEG parameters of a log2-fold change of greater than 1 or less than −1, and a *P* value of less than 0.05, we found two significantly upregulated genes (*Vdr* and *Robo3*) and eight significantly downregulated genes (*Gulo*, *Mef2c*, *Cckbr*, *Slc6a13*, *Crybb3*, *Igfbp7*, *Chrnb4*, and *Abi3bp)* (Fig. 9H). We validated a subset of these DEGs using qPCR and found that elevated PRDM13 significantly increases and decreases specific amacrine cell-related genes, such as *Mef2c*, *Cckbr*, *Vdr*, and *Tpm2*, but not all amacrine cell genes (Fig. 10). Of note, some of these amacrine cell-related genes significantly altered by elevated *PRDM13* have also been shown to be associated with cone photoreceptors, retinal degeneration, and patterning in retinal development.^17^^,^^29^^–^^32^

### No Significant Changes Were Detected in Organs Outside of the Eye for PRDM13-OE Mice Compared With Wildtype Littermates

During our study, we noted that PRDM13-OE mice provided dox for 6 and 10 days had a significant decrease in body weight compared with their wildtype littermates also provided with dox for the same amount of time (Supplementary Fig. S8). However, PRDM13-OE mice recovered and matched the body weight of their wildtype littermates within 3 weeks after the removal of dox. To test for additional adverse systemic effects of elevated PRDM13, histopathology was performed on wildtype and PRDM13-OE mice on day 10 of dox delivery, when the PRDM13-OE mice present with the significantly reduced body weight (Supplementary Table S1). No morphological abnormalities were detected for the brain, heart, lung, liver, kidney, spleen, pancreas, intestines, or stomach.

## Discussion

Photoreceptors are required for visual processing, and the loss of these cells leads to retinal degenerative disease and blindness. However, the mechanisms governing photoreceptor health, maintenance, and function are not understood fully, limiting progress toward treatments for retinal degenerative disorders. To investigate mechanisms regulating photoreceptor health, we developed a mammalian model to induce controlled expression of a transcriptional regulator that has been linked to retinal development and retinal disease: PRDM13. By inducing expression of human *PRDM13* in the mature retina, we are able to assess the impact of exogenous human *PRDM13* expression without affecting endogenous mouse *Prdm13* expression. We discovered that systemic overexpression of *PRDM13* reduces photoreceptor function and survival.

One limitation of our current study is the use of a Tet-On system, which has been known to allow some leaky expression of the insert gene without tetracycline activation, and this is something we see at a minimal extent in our system.^33^^,^^34^ However, we accounted for this limitation by including untreated and dox-treated PRDM13-OE mice and their wildtype littermates in all experiments. We also did not note significant phenotypic changes in untreated PRDM13-OE mice compared with wildtype littermate controls. We did find that dox alone altered some visual function tests, such as the c-wave ERG, as well as various transcripts within the neural retina. To reduce the confounding effect of dox on our data results, we compared our PRDM13-OE mice to their wildtype littermates that also received dox for similar durations of time. Overall, this confounding effect of dox alone emphasizes that researchers need to be cautious when providing dox to mice and assessing visual outcomes.

This work focused primarily on *PRDM13* gene expression, because we found currently available antibodies for protein detection to be unreliable. We tested various antibodies for PRDM13 on both western blot and immunohistochemistry, and these antibodies were unable to detect protein in wildtype mouse retinas where endogenous PRDM13 should be detected in the amacrine cells. To bypass this issue, we confirmed in embryonic retinas that our elevated human *PRDM13* represses its known targets in development, indicating that it is functioning as expected. We also confirmed activation of the negative feedback loop present during development which reduced endogenous mouse *Prdm13*.

Although our model system causes a nonphysiological elevation of the *PRDM13* gene systemically in the presence of dox, it allows us to probe for the effects of *PRDM13* in adult retinal tissue without the confounding effects of the feedback loop present in development. Thus, in the current study, we cannot rule out cell nonautonomous versus cell-autonomous effects, but this model can now provide important insights into the regulatory targets and retinal cell types affected by PRDM13, which are currently unknown. Once known, dysregulation of PRDM13 can be targeted specifically to certain retinal cell types and investigated both in the mature and developing retina in future studies.

In addition, although our system can cause effects from *PRDM13* that would not occur under normal physiological conditions, we did not detect major abnormalities outside of the neural retina, except for a reduction in body weight that recovered after removal of dox (Supplementary Fig. S8, Supplementary Table S1). However, it remains possible that elevated PRDM13 could cause smaller, but significant, alterations in other organs and cells outside of the retina. Future studies can explore the effects of dysregulated PRDM13 on other cells and organs, and how these may impact the retina.

In our mouse model, the removal of excess *PRDM13* expression halted the retinal degenerative phenotype and allowed for some restoration of photoreceptor function. ERG analysis in the PRDM13-OE mice treated with dox for 6 days showed a significant decrease in visual function, but cell death was not detected until after 10 days of dox delivery. This finding suggests that photoreceptor neurons that did not succumb to cell death may restore their functional output when *PRDM13* expression is returned to endogenous levels. We also determined that glial activation occurred after the overexpression of *PRDM13*. Microglia activation and migration to the subretinal space occurred 1 week after *PRDM13* was no longer elevated, before reducing over the next month, and Müller glial activation was noted 3 weeks after *PRDM13* was no longer elevated but was no longer detected two months later. This likely reflects why function of remaining photoreceptors remained low until the second month of ERG testing but then recovered at that time.

In addition, transcriptional dysregulation was still detected by qPCR in the retinas of PRDM13-OE mice for *Gnb3*, a gene responsible for the retina response to light stimuli, at 1 week after the removal of dox and loss of aberrant *PRDM13* expression.^35^ We also found a significant difference in GNB3 protein at the same time in which ERG analysis was performed in Fig. 2, 10 days of dox delivery and 1 week of recovery, which could indicate that the retina was unable to appropriately respond to light stimuli during ERG. After an additional 2 weeks of recovery, the PRDM13-OE retinas no longer showed a difference in GNB3 protein compared with wildtype controls, and a significant increase in ERG amplitudes was detected in PRDM13-OE mice at this time. In support of this, the delay in *Htra3*, *Pde6c*, and *Gnb3* expression correlates with the timing of glial activation. The later recovery of these gene expression levels and reduction of glial activation likely contributes to the ERG recovery.

The downregulation of genes associated with photoreceptor health, visual perception, and retinal development after elevation of *PRDM13* suggests that the photoreceptor degeneration we discovered is likely due to *PRDM13* activity and not toxicity. The fact that the only DEGs significantly associated with biological processes were those that were downregulated when *PRDM13* expression was increased further supports its role as a transcriptional repressor in the retina, as it is known to be in the dorsal neural tube.^11^^,^^12^ Furthermore, prior studies investigating the loss of *Prdm13* in the mammalian retina have shown that *Prdm13* affects the development of GABAergic and glycinergic amacrine cells^13^ and that PRDM13 is necessary for the formation of the early B-cell factor 3–positive amacrine cell subtype.^14^ Intriguingly, we found that increased *PRDM13* expression significantly dysregulated *Robo3*, *Mef2c*, *Crybb3*, and *Gulo*, among others—genes marking subpopulations of amacrine cells.^17^ Of note, some of these significantly dysregulated genes associated with subpopulations of amacrine cells are also known to affect photoreceptors during development, such as *Mef2c*.^29^^,^^30^ Overall, these transcriptional data suggest a novel role for *PRDM13* in the maintenance of both photoreceptors and amacrine cell subpopulations within the retina, warranting future follow-up studies.

We also discovered that genes associated with inherited retinal dystrophies were downregulated in the retina when *PRDM13* expression was elevated. One of these genes, Nuclear Receptor Subfamily 2 Group E Member 3 (*Nr2e3*) is expressed in both developing and mature retinal tissue.^15^ During development, *Nr2e3* is a key regulator of photoreceptor specification and has been linked to multiple inherited retinal diseases such as RP and clumped pigmentary retinal degeneration.^16^ Studies of *Nr2e3^rd7/rd7^* mice, which have a loss of *Nr2e3* expression, found altered expression of genes important for phototransduction and photoreceptor health within the retina.^16^^,^^36^^,^^37^ This includes direct regulation of *Opn1sw*, *Gnb3*, and *Gnat2*, and indirect regulation of *Guaca1a*, *Cryaa*, and *Crygb.*^15^ Each of these genes were found to be downregulated in our RNA sequencing dataset with elevated *PRDM13* expression. We further validated that NR2E3 protein is reduced when *PRDM13* is elevated (3 days of dox), and remained reduced after the removal of dox. NR2E3 is expressed in the photoreceptor ONL,^38^ and the decrease over time is likely due to photoreceptor degeneration in our PRDM13-OE retinas. We also discovered an increase in *Pde6c* after elevation of *PRDM13*, which has been shown in the *Nr2e3^rd7/rd7^* mice to be mis-expressed in rod photoreceptors.^27^^,^^28^ Although immunostaining showed an increase in PDE6C in the PRDM13-OE retinas compared with wildtype littermates, it did not appear to be expressed in the rhodopsin^+^ rod photoreceptors. Future studies are needed to determine whether NR2E3 is directly or indirectly interacting with PRDM13.

A recent study investigating PRDM13’s role in eye field specification showed that PRDM13 is able to activate the WNT/β-catenin signaling pathway at the initiation of retinal differentiation using mouse retinal organoids.^39^ In our current study, GO analysis for biological processes using the downregulated DEGs from our neural retina RNA sequencing dataset returned WNT signaling as a significant biological process. The difference between activation of the WNT/β-catenin pathway during retinal development versus repression of this pathway in the mature retina suggests that PRDM13 has different regulatory actions during retinal development and within the mature retina. However, our data further strengthen the association between PRDM13 and the regulation of WNT signaling within the retina. This finding stresses the need for continued investigation into PRDM13’s direct gene regulatory targets to define its role during retinal development, maturation, and maintenance.

Overall, our mouse model has shown that *PRDM13* plays an important role in the mammalian retina, particularly for phototransduction and photoreceptor health. This model system can now be used to further elucidate the direct and indirect targets of PRDM13 that impact photoreceptor maturation, maintenance and function during mammalian retinal development and in retinal degeneration.

## Supplementary Material

Supplement 1

Supplement 2

Supplement 3
