# Supplementary material for: Elevated PRDM13 Disrupts Photoreceptor Function and Survival in the Mammalian Retina
Source: Invest Ophthalmol Vis Sci. 2025 Aug 18;66(11):38. doi: 10.1167/iovs.66.11.38 (PMC12366859; doi:10.1167/iovs.66.11.38)
Supplement: Supplement 1 [file iovs-66-11-38_s001.pdf]

TITLE: Elevated PRDM13 disrupts photoreceptor function and survival in the mammalian retina

AUTHORS: Emily R. Nettesheim[1], Ashley A. Rowe[1], Tiffany Yee[1], Ahmed Alshaikhsalama[1], Tyler Cepica[1], Vijaya Dutt[1], Samita S. Virani[1], Glen Wickersham[1], Vinit B. Mahajan[2,3], Kent W. Small[4], Katherine J. Wert[1,5,6,7*]

AFFILIATIONS:

[1] Department of Ophthalmology, University of Texas Southwestern Medical Center, Dallas, TX, 75390, USA

[2] Molecular Surgery Laboratory, Byers Eye Institute, Department of Ophthalmology, Stanford University, Palo Alto, CA, USA

[3] Veterans Affairs Palo Alto Health Care System, Palo Alto, CA, USA

[4] Macula and Retina Institute, Molecular Insight Research Foundation, Glendale, CA 91203 USA

[5] Department of Molecular Biology, University of Texas Southwestern Medical Center, Dallas, TX, 75390, USA

[6] Peter O'Donnell Jr. Brain Institute, University of Texas Southwestern Medical Center, Dallas, TX, 75390, USA

[7] Hamon Center for Regenerative Science and Medicine, University of Texas Southwestern Medical Center, Dallas, TX, 75390, USA

* Corresponding Author

CORRESPONDING AUTHOR: Katherine J. Wert, 5323 Harry Hines Blvd. E07.224A, Department of Ophthalmology, University of Texas Southwestern Medical Center, Dallas, Texas 75390, USA. Phone: 214.648.6192; Email: Katherine.Wert@UTSouthwestern.edu.

Supplemental File Word Count: 236.

Funding: KJW is supported by the National Eye Institute (R21EY034597, R01EY037077, R01EY036043, P30EY030413), the Department of Ophthalmology Pilot Grant Award funds, a Department Challenge Grant from the Research to Prevent Blindness, Inc., and gifted funds from the Van Sickle Family Foundation. ERN, AAR, and TY are supported by the National Institute of Health (5T32GM131945). AAR is supported by the National Eye Institute (1F31EY036730). TY is supported by the UT Southwestern Medical Center Hamon Center for Regenerative Science and Medicine Fellowship. VD and SSV are supported by the UT Southwestern Medical Center Green Fellowship Program. GW is supported by the UT Southwestern Medical Center Summer Undergraduate Research Fellowship Program. VBM is supported by NIH grants (R01EY031952, R01EY031360, R01EY030151, and P30EY026877), the Stanford Center for Optic Disc Drusen, and Research to Prevent Blindness, New York, New York.

CONFLICT OF INTEREST STATEMENT. Kent W. Small holds United States Patent No: US 10,973,855 B2, April 13, 2021, Methods for Treating Macular Degeneration. All other authors have declared that no conflict of interest exists.

SUPPLEMENTAL METHODS

Doxycycline (dox) administration during embryonic development. Timed matings were set-up between wildtype female mice and male PRDM13-OE mice. Embryonic day (E) 0.5 was determined by the presence of a copulatory plug. When embryos reached E13.5, the pregnant female was given water containing 0.5mg/mL dox (Sigma-Aldrich, D9891-100G) and 10% sucrose (Sigma-Aldrich, S0389-1KG) for 24 hours *ad libitum* in a red tinted water bottle.

C-wave electroretinography (ERG). C-wave ERG recordings were collected on the Celeris ERG system from Diagnosys, LLC (Lowell, MA, USA) following previously published methods.[19] An average of five sweeps were recorded per eye.

Histopathology. Mice were taken to the University of Texas Southwestern Medical Center Animal Resource Center (ARC) Diagnostics Laboratory, where they were assessed for any gross phenotypic abnormalities, followed by collection of the tissues of interest – brain, heart, lung, liver, kidney, spleen, pancreas, intestines, and stomach. Tissues were dehydrated using alcohol, paraffin embedded and sectioned using a microtome. Sections were then stained with hematoxylin and eosin (H&E) and sent for analysis by the ARC Diagnostic Laboratory's pathologist.

Body weight measurement. Female PRDM13-OE and wildtype littermates were given 2mg/mL dox + 10% sucrose in a red tinted water bottle *ad libitum* for 0-10 days. Body weight, in grams, was recorded on a standard lab balance one-week post-removal of dox (1 month of age), three weeks post-removal of dox (2 months of age), and seven weeks post-removal of dox (3 months of age).

FIGURES

Supplemental Figure 1

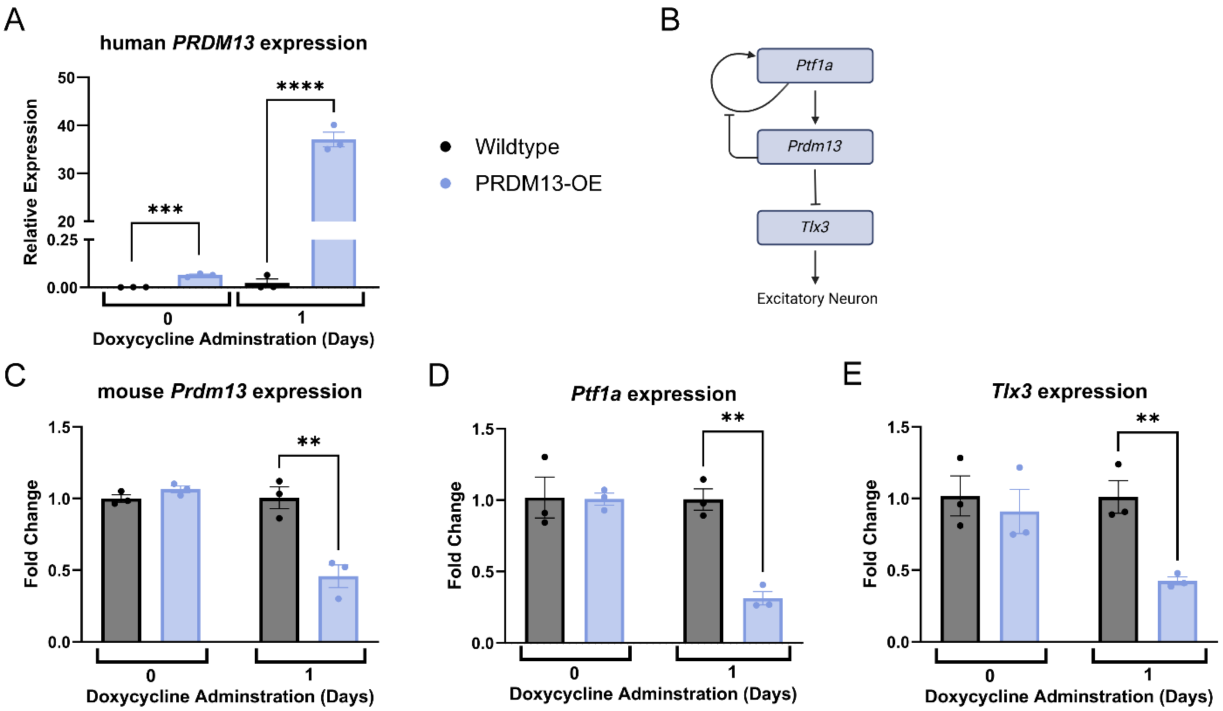

Figure S1. Exogenous *PRDM13* expression leads to inhibition of *Ptf1a*, *Prdm13* and *Tlx3* expression in the developing retina. Pregnant female mice carrying litters of wildtype and PRDM13-OE embryos were either untreated or treated with 0.5mg/mL dox and 10% sucrose for 24-hours from E13.5 to E14.5. **(A)** Embryonic retina samples were analyzed by qPCR for human *PRDM13* expression. **(B)** Schematic of known gene regulation up- and down-stream of *Prdm13*. Relative expression of **(C)** *Prdm13*, **(D)** *Ptf1a*, and **(E)** *Tlx3* in the same embryonic retinal tissue used to assess *PRDM13* expression in **(A)**. Data was analyzed by unpaired t-test between the wildtype and PRDM13-OE groups that received the same amount of dox. Error bars = SD. **, p<0.01; ***, p<0.001; ****, p<0.0001. N = 3 biological replicates, each an average of 3 technical replicates.

Supplemental Figure 2

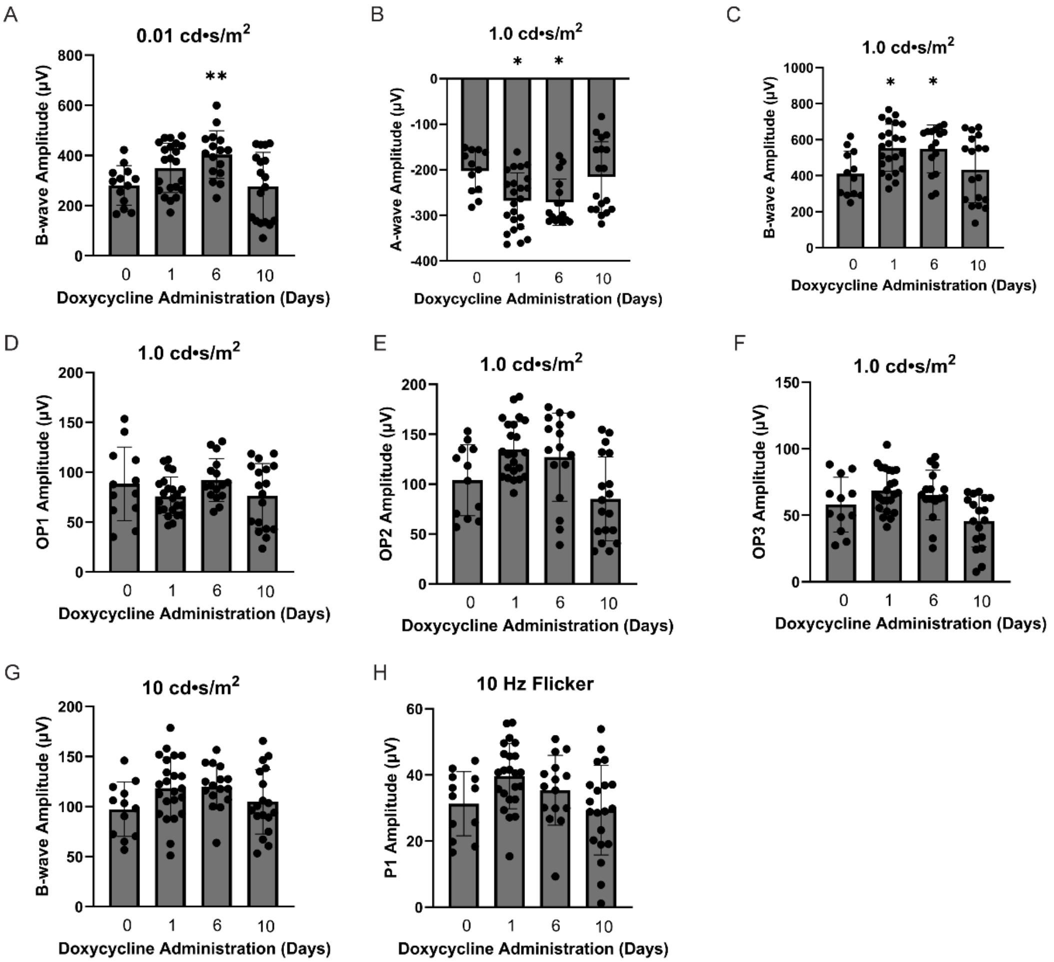

Figure S2. Administration of dox does not cause adverse visual function outcomes in wildtype mice. Wildtype mice were provided dox for zero, one, six or ten days beginning at post-natal day (P)28. Scotopic and photopic ERG was performed one-week post-removal of dox. Amplitudes for scotopic **(A)** 0.01 cd•s/m^2 b-wave, **(B)** 1.0 cd•s/m^2 a-wave, **(C)** 1.0 cd•s/m^2 b-wave, **(D)** 1.0 cd•s/m^2 oscillatory potential (OP)1, **(E)** OP2, **(F)** OP3, and photopic **(G)** 10 cd•s/m^2 b-wave and **(H)** 10 Hz flicker. Data was analyzed by one-way ANOVA with Dunnett's multiple comparisons test. Error bars = SD. Significance is shown between the wildtype group without dox to each dox group. *, $p<0.05$; **, $p<0.01$. N ≥ 13 eyes.

Supplemental Figure 3

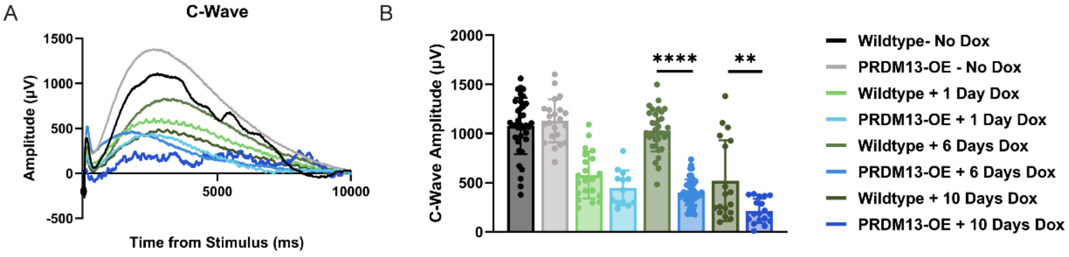

Figure S3. Retinal pigment epithelial (RPE)/photoreceptor function is reduced with elevated *PRDM13* for greater than six days. PRDM13-OE mice and wildtype littermates were untreated or provided dox for one, six or ten days beginning at P28. Mice underwent c-wave ERG at two months of age. **(A)** Representative traces from a single mouse, and **(B)** ERG amplitudes for the cohort of mice. Data was analyzed by one-way ANOVA with Tukey's multiple comparison's test. Error bars = SD. Significance is shown for PRDM13-OE mice compared to their respective wildtype control for each group. **, $p<0.01$; ****, $p<0.0001$. N ≥ 13 eyes.

Supplemental Figure 4

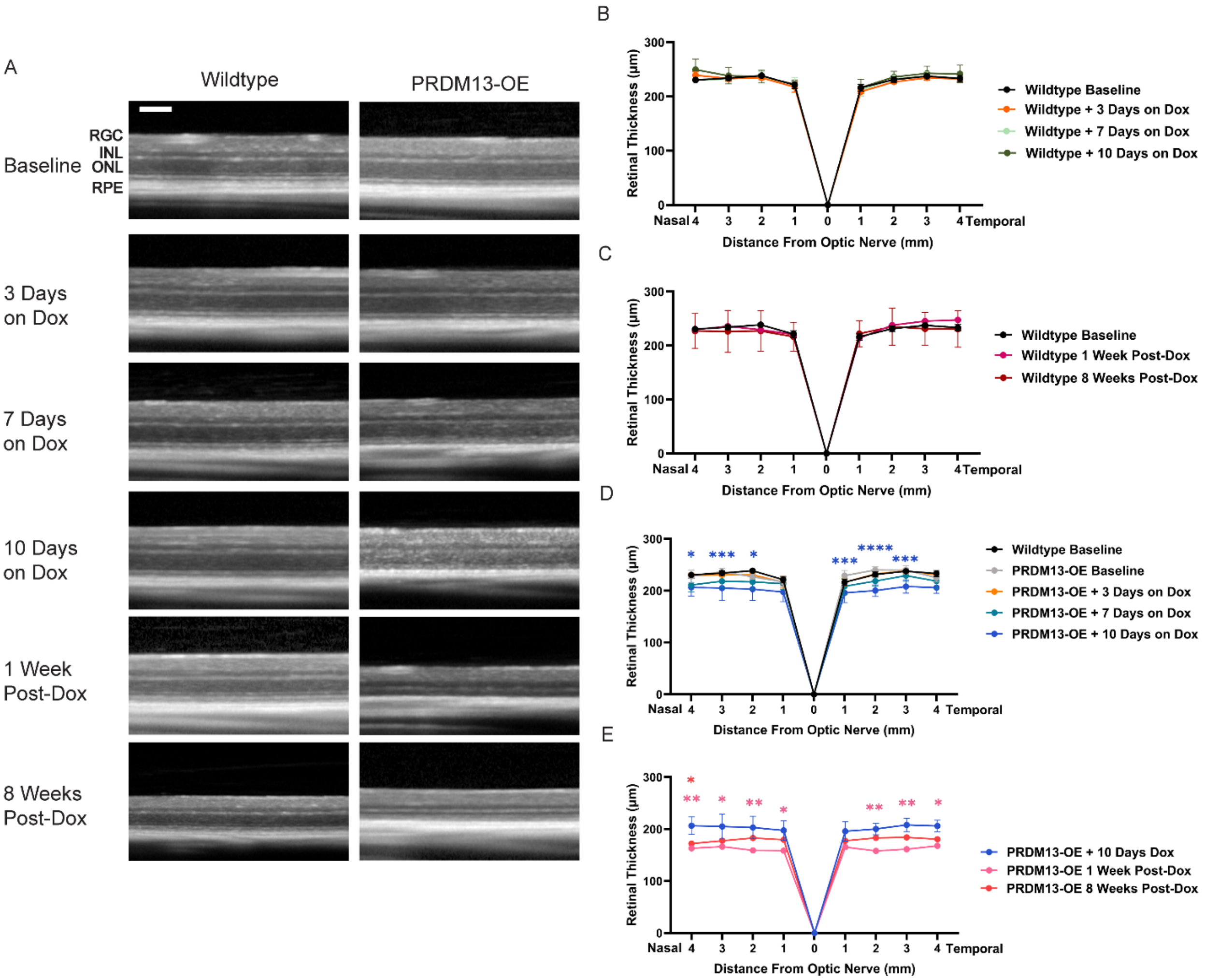

Figure S4. Elevated *PRDM13* causes retinal degeneration but stabilizes when *PRDM13* is no longer dysregulated. (A) Representative OCT images collected before, during and after administration of dox in the same wildtype and PRDM13-OE mice over time. Scale bar = 1µm. RGC, retinal ganglion cells; INL, inner nuclear layer; ONL, outer nuclear layer; RPE, retinal pigment epithelium. Total retinal thickness (TRT) measurements were collected spanning 1mm distances from the optic nerve head (ONH) for **(B-C)** wildtype and **(D-E)** PRDM13-OE mice before, during, and after dox administration. Data was analyzed by two-way ANOVA with Tukey's multiple comparison's test. Error bars = SD. Significance is shown against the **(B-D)**

baseline group or **(E)** PRDM13 + 10 Days Dox, with asterisks colored to match the experimental group they represent. $N \geq 3$ mice per group. *, $p<0.05$; **, $p<0.01$; ***, $p<0.001$; ****, $p<0.0001$.

Supplemental Figure 5

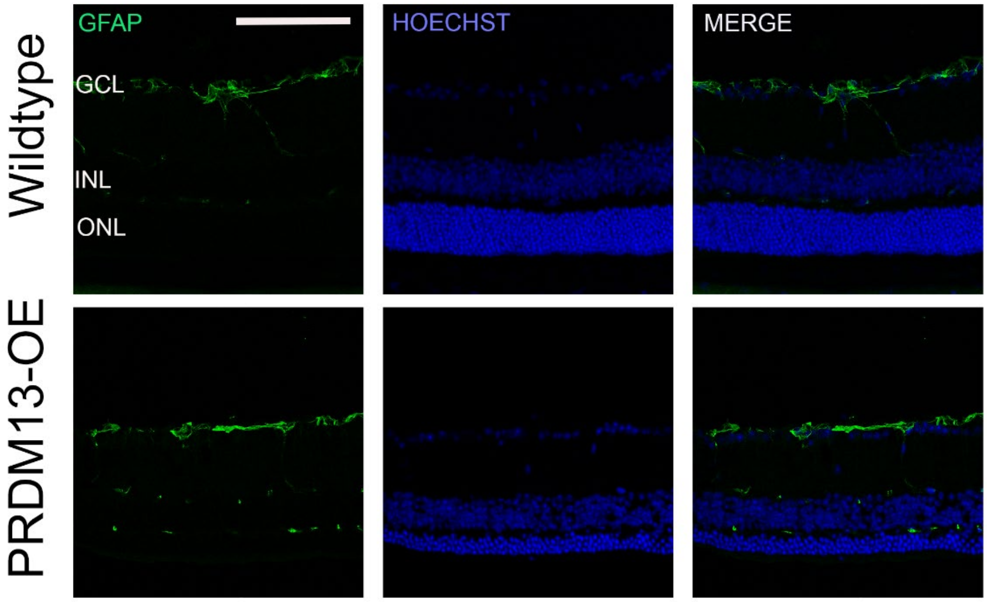

Figure S5. Glial activation is no longer detectable three months post-removal of dox. Representative retinal sections for wildtype and PRDM13-OE mice which had received dox for ten days beginning at P28 and immunostained for glial fibrillary acidic protein (GFAP, green) three months post-removal of dox. Three biological replicates with three technical replicates each were analyzed. Scale bar = 100µm. Hoescht, nuclei (blue). GCL, ganglion cell layer; INL, inner nuclear layer; ONL, outer nuclear layer.

Supplemental Figure 6

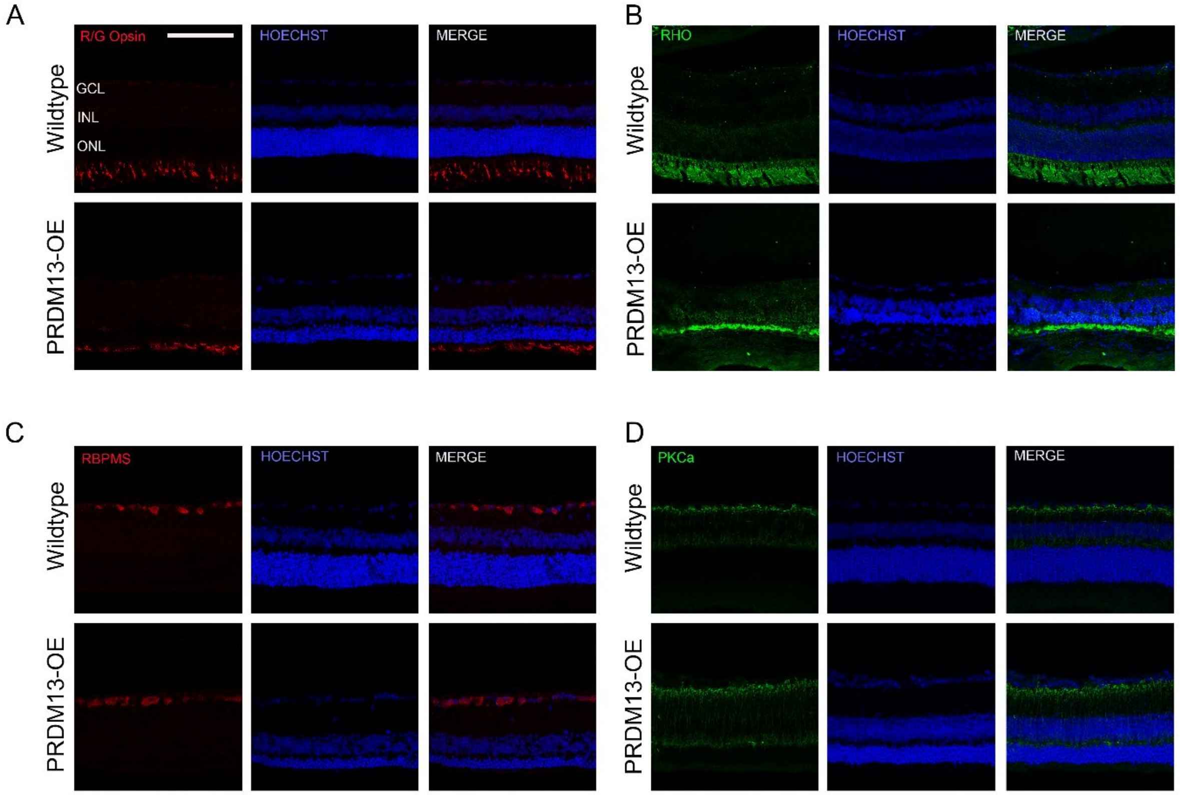

Figure S6. Elevated *PRDM13* does not cause large-scale morphological abnormalities of ganglion or bipolar cells. Representative retinal sections for PRDM13-OE mice and wildtype littermates provided dox beginning at P28 for ten days, then retinas were collected eleven weeks post-removal of dox. Retinas were stained for **(A)** R/G opsin (red), **(B)** Rhodopsin (green), **(C)** RBPMS (red) or **(D)** PKCα (green) and Hoechst for nuclei (blue). Three biological replicates with three technical replicates each were analyzed. Scale bar = 100μm. GCL, ganglion cell layer; INL, inner nuclear layer; ONL, outer nuclear layer.

Supplemental Figure 7

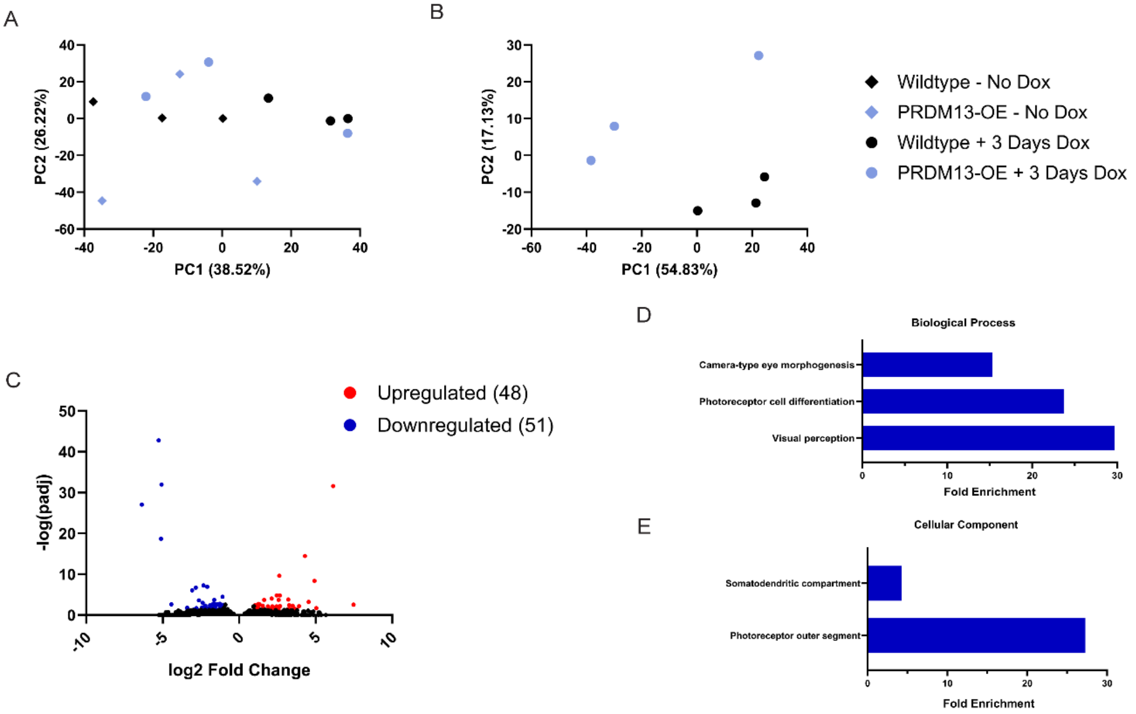

Figure S7. Elevated *PRDM13* minimally alters gene expression of the RPE, choroid, and sclera. Using the same samples for neural retina dissection in Figure 6 (PRDM13-OE mice and wildtype littermates that were untreated or treated with dox for three days beginning at P28), eye cups containing the choroid, sclera and retinal pigment epithelium were collected, RNA was isolated, and RNA-sequencing was performed. **(A)** Principal component analysis (PCA) plot for all biological replicates and control groups. **(B)** PCA plot for PRDM13-OE and wildtype littermates treated with dox. **(C)** PRDM13-OE and wildtype retinas that both received dox were compared for differential gene expressions. Upregulated (red, 48 genes) and downregulated (blue, 51 genes) genes were determined using a log2 fold change of greater than 1 or less than -1 and an adjusted p-value < 0.05. GO analysis for significantly downregulated genes highlights significant **(D)** Biological Processes and **(E)** Cellular Components affected by elevated *PRDM13*.

Supplemental Figure 8

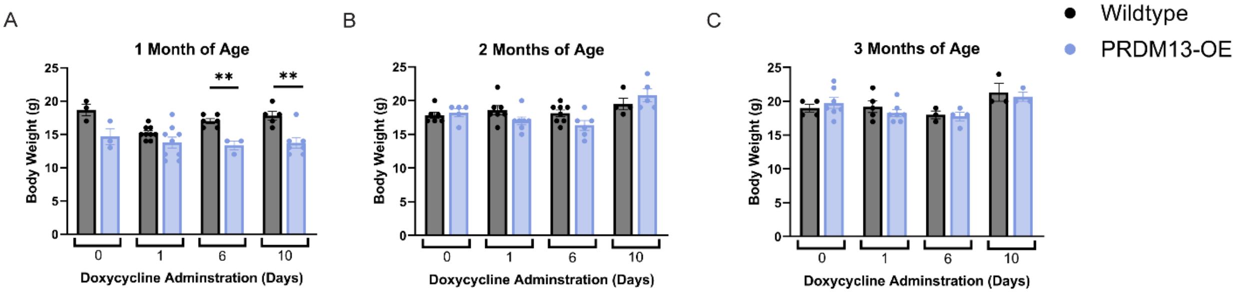

Figure S8. Elevated *PRDM13* for six to ten days causes a transient reduction in body weight. Female PRDM13-OE and wildtype littermates were provided dox for 0-10 days beginning at P28. Mice were weighed **(A)** one week post-removal of dox (1 month of age), **(B)** three weeks post-removal of dox (2 months of age), and **(C)** seven weeks post-removal of dox (3 months of age). Data was analyzed by unpaired t-test between wildtype and PRDM13-OE mice who had received the same amount of dox. Error bars = SD. **, $p<0.01$. $N \geq 3$ mice.

SUPPLEMENTAL TABLES

Supplemental Table 1

Tissue	Wildtype + 10 Days Dox (N=3)	PRDM13-OE + 10 Days Dox (N=4)
Brain	Morphologically normal (N=3)	Morphologically normal (N=4)
Heart	Morphologically normal (N=3)	Morphologically normal (N=3)<br>Small focal area of myofiber mineralization (N=1)
Lung	Morphologically normal (N=3)	Morphologically normal (N=2)<br>Vessels are diffusely congested; Multifocal alveoli contain hemorrhage; occasional alveoli contain alveolar macs (N=2)
Liver	Morphologically normal (N=3)	Morphologically normal (N=3)<br>Multifocal infiltrates with small numbers of mixed inflammatory cell infiltrates with hepatocellular necrosis (micro-abscess) in (N=1)
Kidney	Morphologically normal (N=3)	Morphologically normal (N=4)
Spleen	Morphologically normal (N=3)	Morphologically normal (N=4)
Pancreas	Morphologically normal (N=2)<br>Scattered apoptosis of exocrine cells but no necrosis or atrophy; some mitosis (N=1)	Morphologically normal (N=3)<br>Scattered apoptosis of exocrine cells but no necrosis or atrophy; some mitosis (N=1)
Intestines	Morphologically normal (N=2)<br>Mild multifocal hypertrophy of mucous cells in colon; however, no mucosal lumen (N=1)	Morphologically normal (N=4)
Stomach	Morphologically normal (N=3)	Morphologically normal (N=3)<br>Mild focal hyperplasia of non-glandular epithelium (N=1)

Table S1. Histopathology shows no detectable adverse pathology following ten days of elevated *PRDM13*. PRDM13-OE mice and wildtype littermates were provided dox in the drinking water beginning at P28 for ten days. Samples were collected on the tenth day, and tissues were analyzed for gross abnormalities by eye and using H&E-stained sections. Diagnostic results and sample sizes are provided within the table.

Supplemental Table 2

Antibody	Company	Catalogue Number	Dilution	Use
RBPMS	Abcam	ab152101	1:100	IHC
GFAP	Sigma-Aldrich	MAB360	1:100	IHC
IBA1	Abcam	ab178846	1:400	IHC
PKCα	Santa Cruz Biotechnology	SC-8393	1:100	IHC
Calbindin	Thermo Fisher Scientific	702411	1:100/1:1000	IHC/Western Blot
Calretinin	Millipore	MAB1568	1:200/1:1000	IHC/ Western Blot
GAD-6	Developmental Studies Hybridoma Bank	GAD-6	1:100/1:1000	IHC/Western Blot
PNA	Invitrogen	L32459	1:200	IHC
Rhodopsin	Millipore	MABN15	1:150	IHC
PDE6c	US Biological Life Sciences	039807	1:100	IHC
Goat anti-Rabbit Alexa Fluor 594	Thermo Fisher Scientific	A11012	1:1000	IHC
Goat anti-mouse Alexa Fluor 488	Thermo Fisher Scientific	A11001	1:1000	IHC
NR2E3	Proteintech	14246-1-AP	1:1000	Western Blot
GNB3	Proteintech	67497-1-IG	1:1000/1:100	Western Blot/IHC
Vinculin	Invitrogen	14-9777-82	1:1000	Western Blot
IR Dye 800CW Goat anti-mouse	LICORbio	926-32210	1:15000	Western Blot
IR Dye 680RD Goat anti-rabbit	LICORbio	926-68071	1:15000	Western Blot

Table S2. Antibodies. Antibodies used in the manuscript.

Supplemental Table 3

Primer Target	Forward Primer (5'-3')	Reverse Primer (5'-3')
U36b4	CGTCCTCGTTGGAGTGACA	CGGTGCGTCAGGGATTG
Beta-actin	CCAGAAGGACTGTTATGTGGGA	GACTCCGTGTTCAATGGGATAC
PRDM13	GGTACCTTCAAGCTGGGCAA	TAGAAGATCTGTCCTCCGGG
Prdm13	GTGGCAACAATGCACGGAAC	CCTGCGATCTGACAGGTACT
Htra3	CTGCACACCATCCAGGACAT	CAGTGGTTTCCCATGGCTGA
Nxnl2	CCCCTACCGGCATGAACTG	TGCTCTCTGTCACAGCATGG
Gnb3	CATCGCAGCTGAGACAGTGA	AGGCACTTACGAGCAGCTTAG
Prdm1	CGTAGAAAAGGAGGGACCGC	TTGGGGGCAGCCAAGGT
Nr2e3	AACTTCTAGCAAGCAGGCTACC	GCTCATTCTGCACAGCATCTTG
Robo3	GAATCGCCGAGAGGAACCAA	GCTACCAGCGTGTATTGACCT
Tulp1	GCCGAGATTAGCTAGGAAGGAAG	GGCGGGTCGCTGTTTGG
Pde6c	GGGGCAGACCATGTTCACTT	TCTGTCCATCATAGGCTGACTCT
Ptf1a	AGGTTATCATCTGCCATCGAG	GACACAAACTCAAAGGGTGGT
Tlx3	TGTCAACCTAAGCTTGGCCC	GGCCGCCGCTGTGAA
Cckbr	CCAACAAATGTGGTCCGTGC	AAAGCGGAGCCCTAGGTAGA
Mef2c	GAAGGGCCTCAATGGCTGT	GGAGGTGGAACAGCACACAA
Vdr	CAAGGGTTTCTTCAGGCGGA	TCATCTCTCGCTTACGCTGC
Tpm2	GGAAGCCCAAGCGGACAAGTA	TTCTGTGCATAGACTTCATCTTCCA
Ptn	CAAGCCTCAAGCGGAGTCA	GCCCTTTTCCTGGTCCACAG
Npy	TACTCCGCTCTGCGACACTA	GGAAGGGTCTTCAAGCCTTGTT
Th	CACCTATGCACTCACCCGAG	TGCAAGTCCAATGTCCTGGG
Ebf3	GGGAACGGACTGCAAGCTAT	TGAAGTCCGTCCTTGGATGC

Table S3. Primer sequences. Primer sequences used in the manuscript.
