# Supplementary material for: Elevated PRDM13 Disrupts Photoreceptor Function and Survival in the Mammalian Retina
Source: Invest Ophthalmol Vis Sci. 2025 Aug 18;66(11):38. doi: 10.1167/iovs.66.11.38 (PMC12366859; doi:10.1167/iovs.66.11.38)

Western Blots, Raw Images

Groups: 0&3 day dox
Technical replicate: 1
Target: Top blot: Vinculin – Loading Control
Bottom blot: Gnb3
Imaging Type: Licor

Samples:
1. Ladder
2. WT, no dox, biological replicate 1
3. PRDM-OE, no dox, biological replicate 1
4. WT + 3 days dox, biological replicate 1
5. PRDM13-OE + 3 days dox, biological replicate 1
6. Ladder
7. WT, no dox, biological replicate 2
8. PRDM-OE, no dox, biological replicate 2
9. WT + 3 days dox, biological replicate 2
10. PRDM13-OE + 3 days dox, biological replicate 2
11. Ladder
12. WT, no dox, biological replicate 3
13. PRDM-OE, no dox, biological replicate 3
14. WT + 3 days dox, biological replicate 3
15. PRDM13-OE + 3 days dox, biological replicate 3

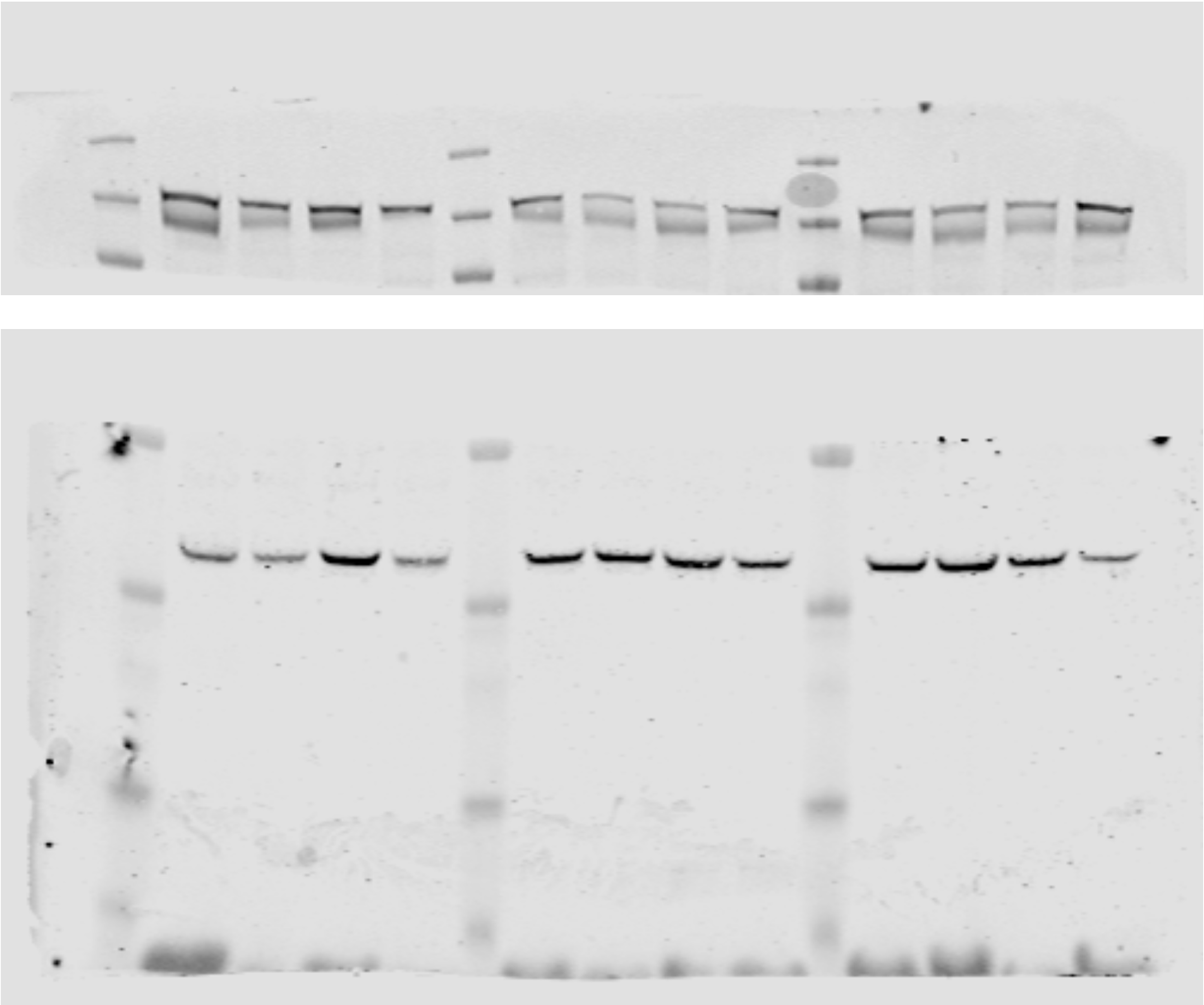

Groups: 10 day dox, 1 and 2 mo
Technical replicate: 1
Target: top Blot: Vinculin – Loading Control
Bottom Blot: Gnb3
Imaging Type: Licor

Samples:
1. Ladder
2. WT + 1 week after a 10 day dox treatment, biological replicate 1
3. PRDM13-OE + 1 week after a 10 day dox treatment, biological replicate 1
4. WT + 3 weeks after a 10 day dox treatment, biological replicate 1
5. PRDM13-OE + 3 weeks after a 10 day dox treatment, biological replicate 1
6. Ladder
7. WT + 1 week after a 10 day dox treatment biological replicate 2
8. PRDM13-OE + 1 week after a 10 day dox treatment, biological replicate 2
9. WT + 3 weeks after a 10 day dox treatment, biological replicate 2
10. PRDM13-OE + 3 weeks after a 10 day dox treatment, biological replicate 2
11. Ladder
12. WT + 1 week after a 10 day dox treatment biological replicate 3
13. PRDM13-OE + 1 week after a 10 day dox treatment, biological replicate 3
14. WT + 3 weeks after a 10 day dox treatment, biological replicate 3
15. PRDM13-OE + 3 weeks after a 10 day dox treatment, biological replicate 3

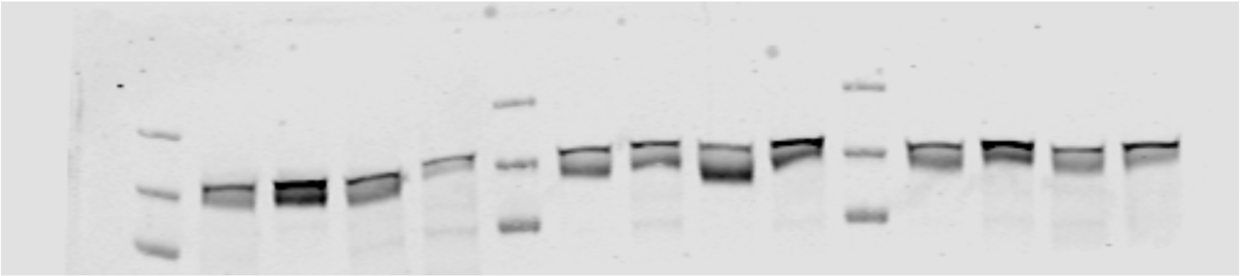

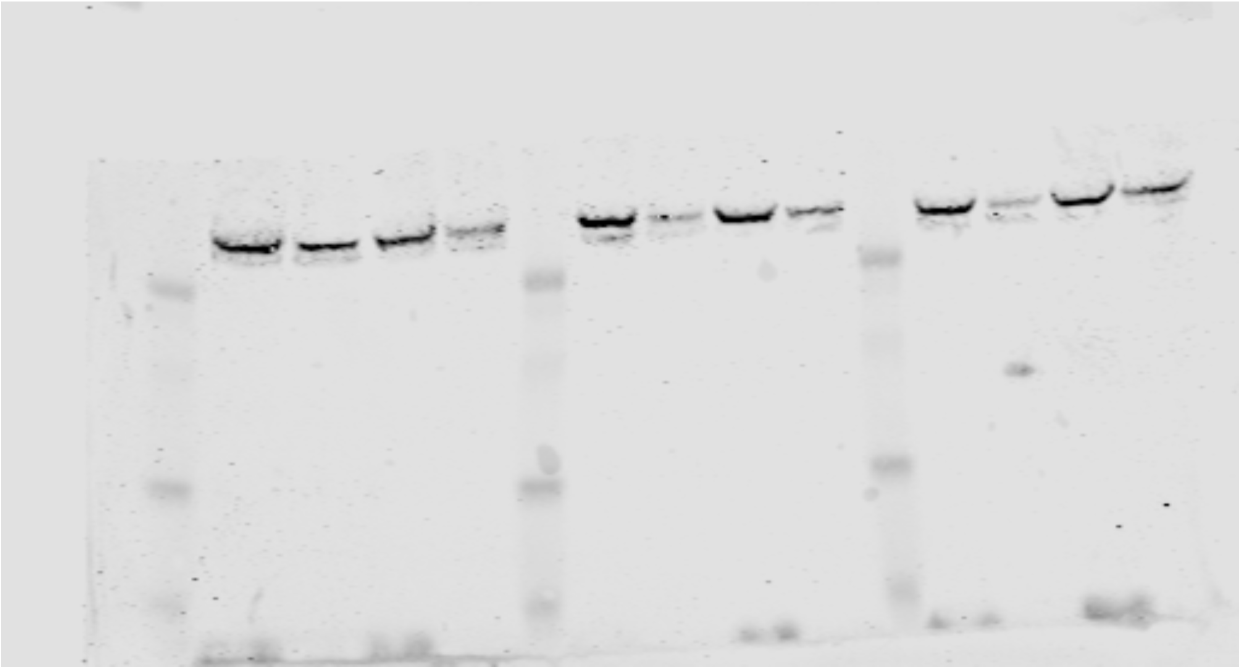

Groups: 0&3 day dox
Technical replicate: 2
Target: Top blot: Vinculin – Loading Control
Middle blot: Nr2e3
Bottom blot: Gnb3
Imaging Type: Licor

Samples:
1. Ladder
2. WT, no dox, biological replicate 1
3. PRDM-OE, no dox, biological replicate 1
4. WT + 3 days dox, biological replicate 1
5. PRDM13-OE + 3 days dox, biological replicate 1
6. Ladder
7. WT, no dox, biological replicate 2
8. PRDM-OE, no dox, biological replicate 2
9. WT + 3 days dox, biological replicate 2
10. PRDM13-OE + 3 days dox, biological replicate 2
11. Ladder
12. WT, no dox, biological replicate 3
13. PRDM-OE, no dox, biological replicate 3
14. WT + 3 days dox, biological replicate 3
15. PRDM13-OE + 3 days dox, biological replicate 3

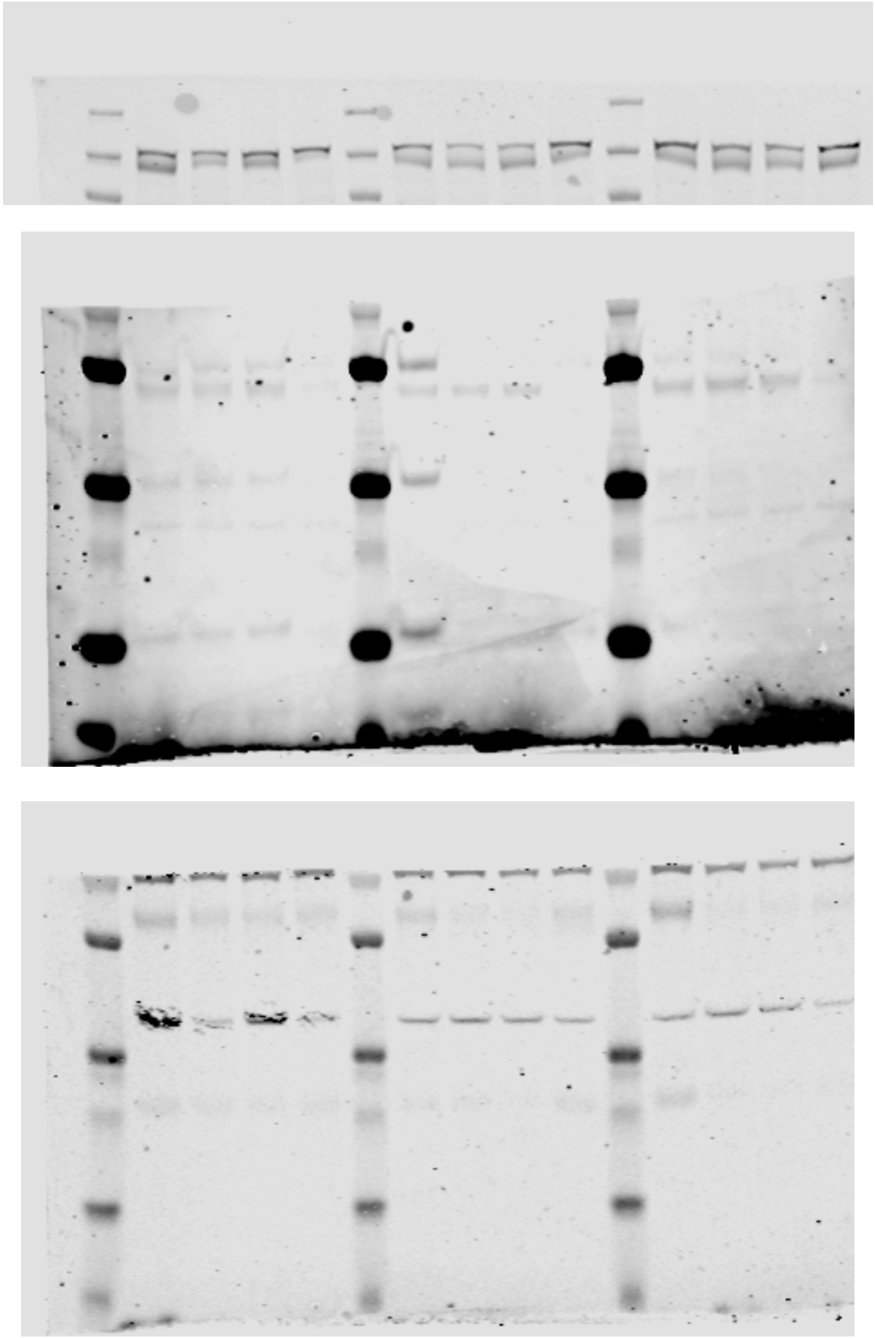

Groups: 10 day dox, 1 and 2 mo
Technical replicate: 2
Target: Top blot: Vinculin – Loading Control
Middle blot: Nr2e3
Bottom Blot: Gnb3
Imaging Type: Licor

Samples:
1. Ladder
2. WT + 1 week after a 10 day dox treatment, biological replicate 1
3. PRDM13-OE + 1 week after a 10 day dox treatment, biological replicate 1
4. WT + 3 weeks after a 10 day dox treatment, biological replicate 1
5. PRDM13-OE + 3 weeks after a 10 day dox treatment, biological replicate 1
6. Ladder
7. WT + 1 week after a 10 day dox treatment biological replicate 2
8. PRDM13-OE + 1 week after a 10 day dox treatment, biological replicate 2
9. WT + 3 weeks after a 10 day dox treatment, biological replicate 2
10. PRDM13-OE + 3 weeks after a 10 day dox treatment, biological replicate 2
11. Ladder
12. WT + 1 week after a 10 day dox treatment biological replicate 3
13. PRDM13-OE + 1 week after a 10 day dox treatment, biological replicate 3
14. WT + 3 weeks after a 10 day dox treatment, biological replicate 3
15. PRDM13-OE + 3 weeks after a 10 day dox treatment, biological replicate 3

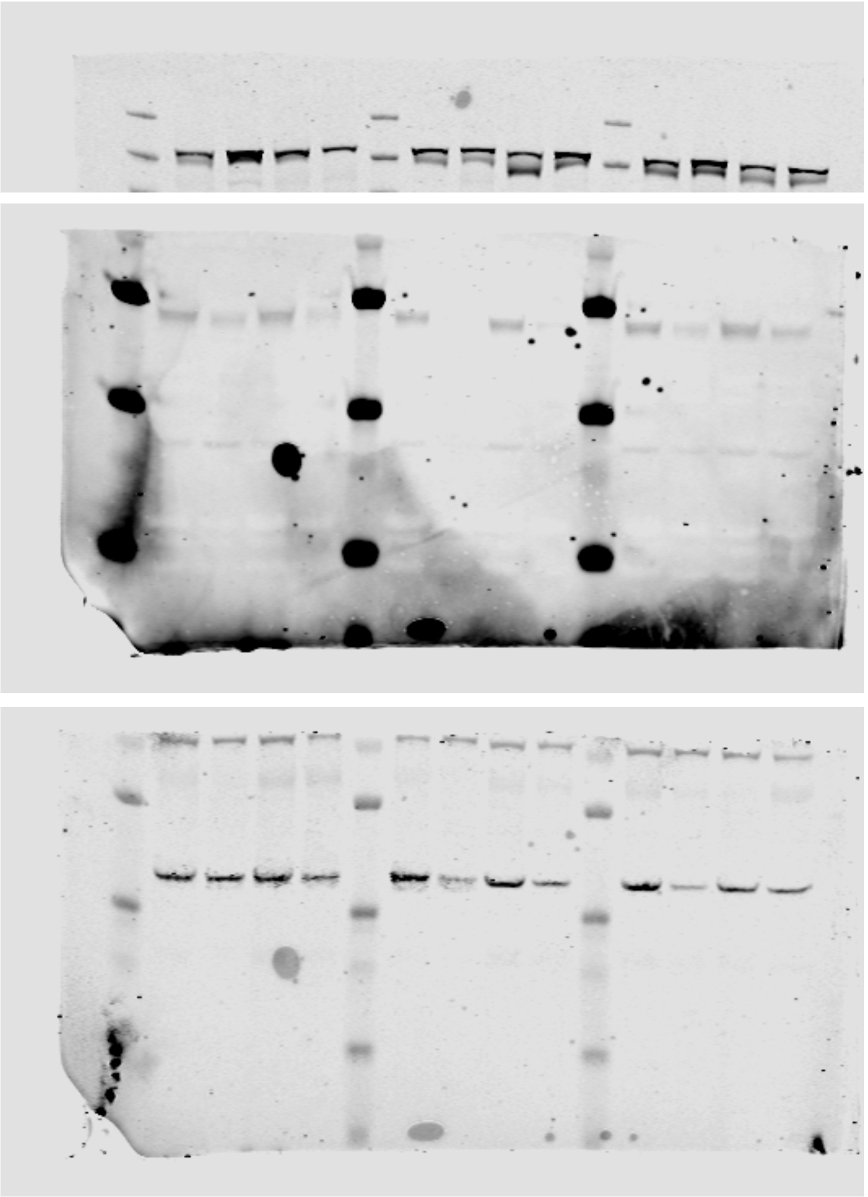

Groups: 0&3 day dox
Technical replicate: 12/13
Target: Top blot: Vinculin – Loading Control
Bottom blot: Nr2e3
Imaging Type: Licor

Samples:
1. Ladder
2. WT, no dox, biological replicate 1
3. PRDM-OE, no dox, biological replicate 1
4. WT + 3 days dox, biological replicate 1
5. PRDM13-OE + 3 days dox, biological replicate 1
6. Ladder
7. WT, no dox, biological replicate 2
8. PRDM-OE, no dox, biological replicate 2
9. WT + 3 days dox, biological replicate 2
10. PRDM13-OE + 3 days dox, biological replicate 2
11. Ladder
12. WT, no dox, biological replicate 3
13. PRDM-OE, no dox, biological replicate 3
14. WT + 3 days dox, biological replicate 3
15. PRDM13-OE + 3 days dox, biological replicate 3

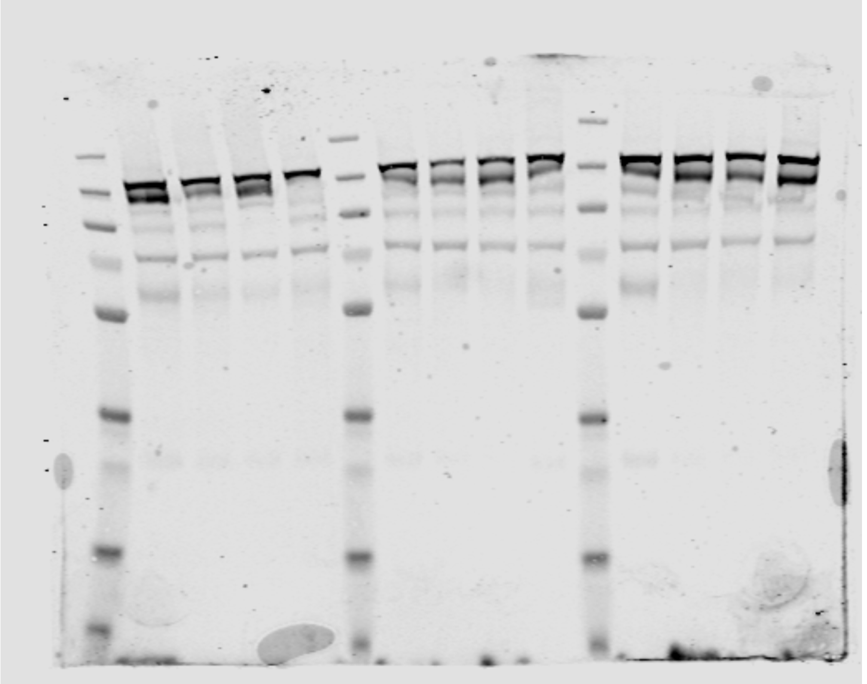

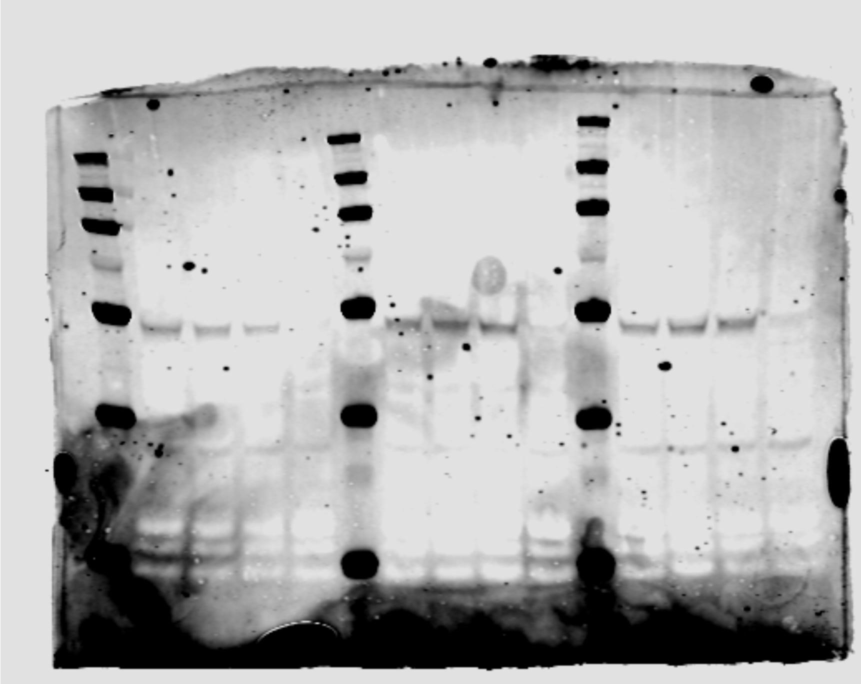

Groups: 10 day dox, 1 and 2 mo
Technical replicate: 3
Target: Top blot: Vinculin – Loading Control
Bottom blot: Nr2e3
Imaging Type: Licor

Samples:
1. Ladder
2. WT + 1 week after a 10 day dox treatment, biological replicate 1
3. PRDM13-OE + 1 week after a 10 day dox treatment, biological replicate 1
4. WT + 3 weeks after a 10 day dox treatment, biological replicate 1
5. PRDM13-OE + 3 weeks after a 10 day dox treatment, biological replicate 1
6. Ladder
7. WT + 1 week after a 10 day dox treatment biological replicate 2
8. PRDM13-OE + 1 week after a 10 day dox treatment, biological replicate 2
9. WT + 3 weeks after a 10 day dox treatment, biological replicate 2
10. PRDM13-OE + 3 weeks after a 10 day dox treatment, biological replicate 2
11. Ladder
12. WT + 1 week after a 10 day dox treatment biological replicate 3
13. PRDM13-OE + 1 week after a 10 day dox treatment, biological replicate 3
14. WT + 3 weeks after a 10 day dox treatment, biological replicate 3
15. PRDM13-OE + 3 weeks after a 10 day dox treatment, biological replicate 3

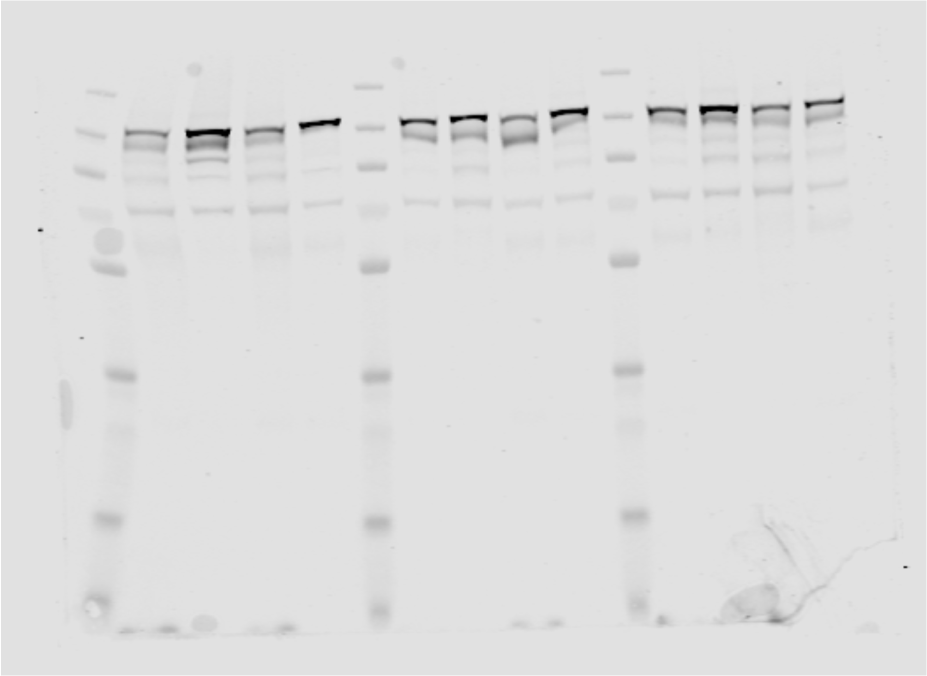

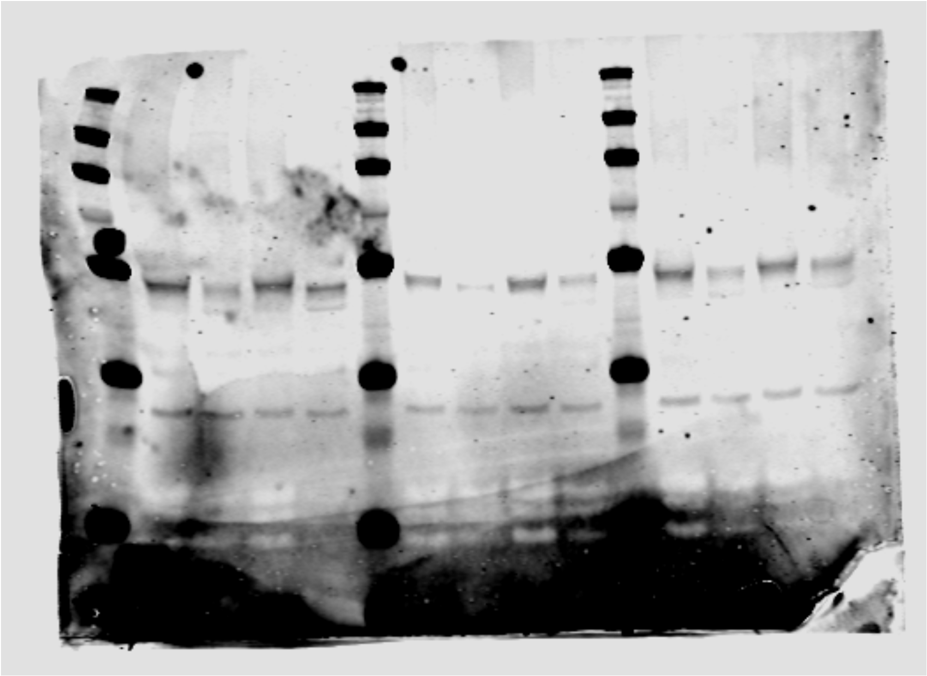

Groups: 0&3 day dox
Technical replicate: 1
Target: Top blot: Robo3
Bottom blot: Gapdh – Loading Control
Imaging Type: ECL

Samples:
1. Ladder
2. WT, no dox, biological replicate 1
3. PRDM-OE, no dox, biological replicate 1
4. WT + 3 days dox, biological replicate 1
5. PRDM13-OE + 3 days dox, biological replicate 1
6. Ladder
7. WT, no dox, biological replicate 2
8. PRDM-OE, no dox, biological replicate 2
9. WT + 3 days dox, biological replicate 2
10. PRDM13-OE + 3 days dox, biological replicate 2
11. Ladder
12. WT, no dox, biological replicate 3
13. PRDM-OE, no dox, biological replicate 3
14. WT + 3 days dox, biological replicate 3
15. PRDM13-OE + 3 days dox, biological replicate 3

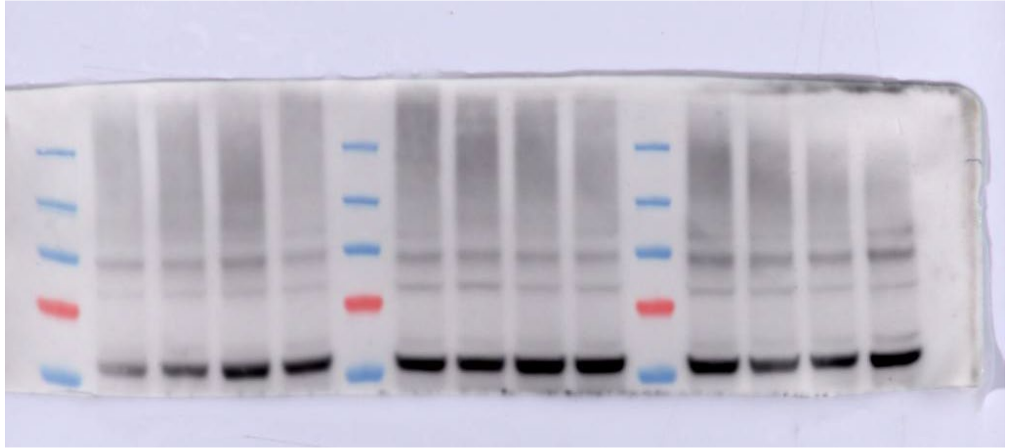

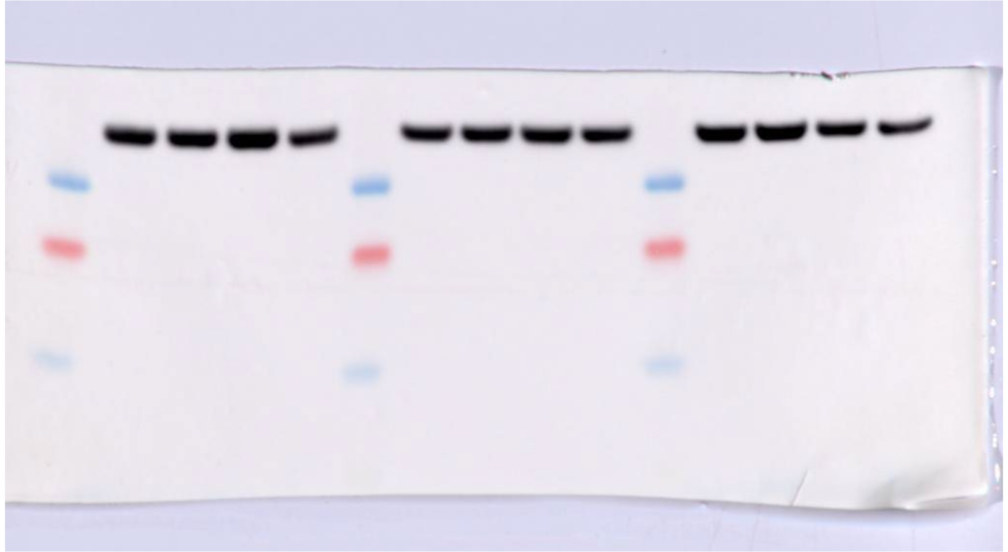

Groups: 0&3 day dox
Technical replicate: 2
Target: Top blot: Robo3
Bottom blot: Gapdh – Loading Control
Imaging Type: ECL

Samples:
1. Ladder
2. WT, no dox, biological replicate 1
3. PRDM-OE, no dox, biological replicate 1
4. WT + 3 days dox, biological replicate 1
5. PRDM13-OE + 3 days dox, biological replicate 1
6. Ladder
7. WT, no dox, biological replicate 2
8. PRDM-OE, no dox, biological replicate 2
9. WT + 3 days dox, biological replicate 2
10. PRDM13-OE + 3 days dox, biological replicate 2
11. Ladder
12. WT, no dox, biological replicate 3
13. PRDM-OE, no dox, biological replicate 3
14. WT + 3 days dox, biological replicate 3
15. PRDM13-OE + 3 days dox, biological replicate 3

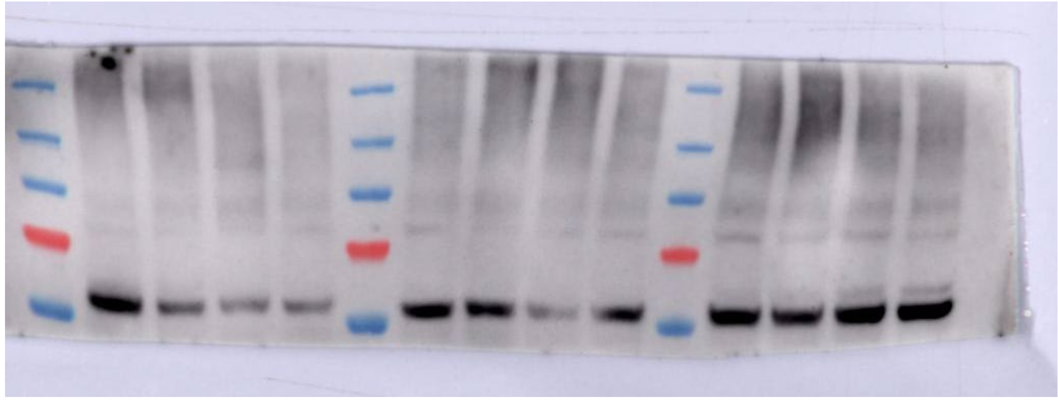

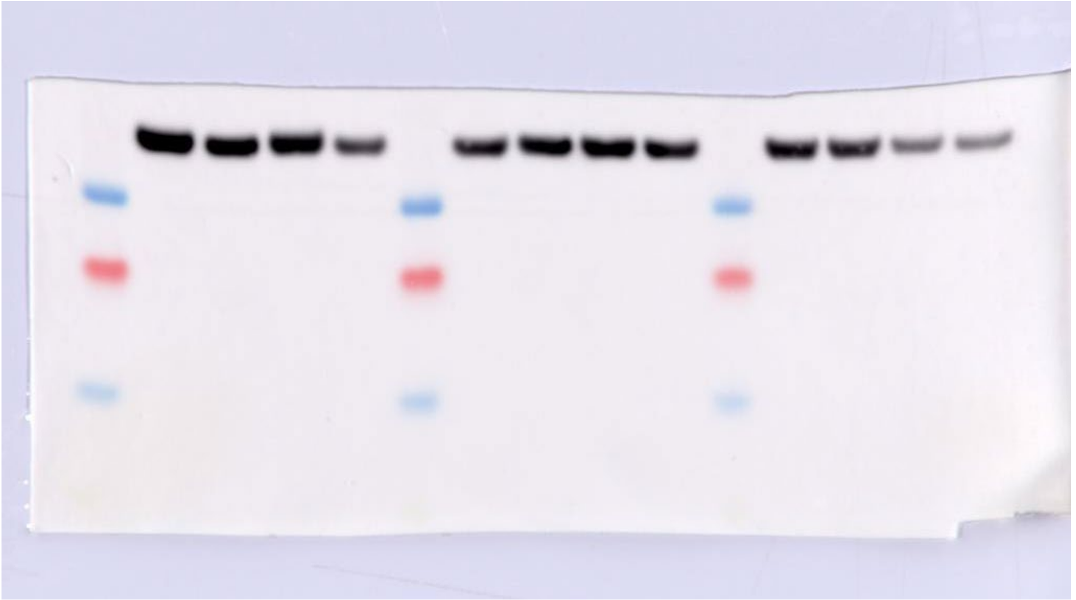

Groups: 10 day dox, 1 and 2 mo
Technical replicate: 1
Target: Top blot: Robo3
Bottom blot: Gapdh – Loading Control
Imaging Type: ECL

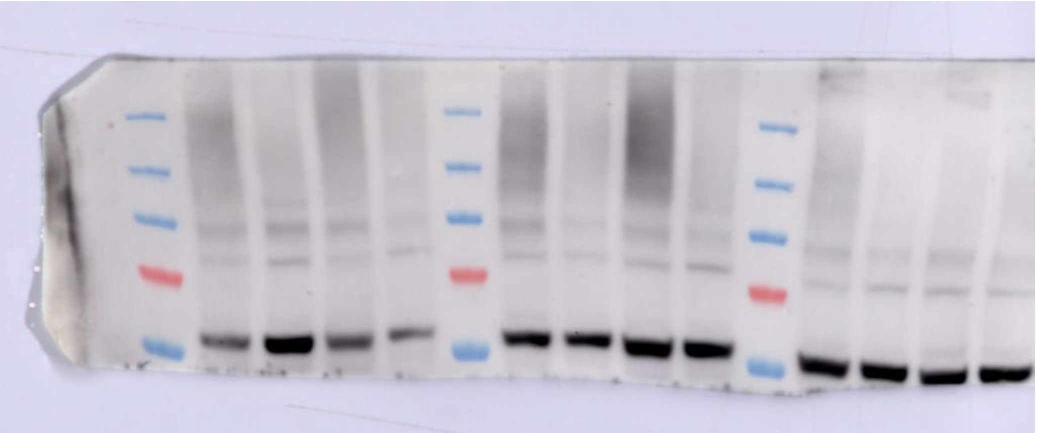

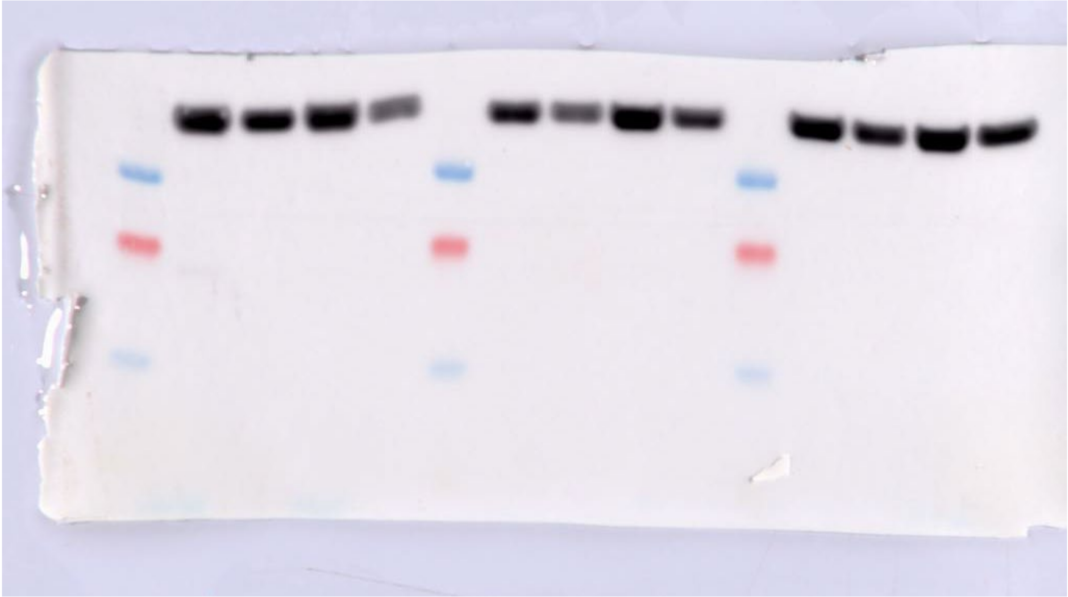

Samples:
1. Ladder
2. WT + 1 week after a 10 day dox treatment, biological replicate 1
3. PRDM13-OE + 1 week after a 10 day dox treatment, biological replicate 1
4. WT + 3 weeks after a 10 day dox treatment, biological replicate 1
5. PRDM13-OE + 3 weeks after a 10 day dox treatment, biological replicate 1
6. Ladder
7. WT + 1 week after a 10 day dox treatment biological replicate 2
8. PRDM13-OE + 1 week after a 10 day dox treatment, biological replicate 2
9. WT + 3 weeks after a 10 day dox treatment, biological replicate 2
10. PRDM13-OE + 3 weeks after a 10 day dox treatment, biological replicate 2
11. Ladder
12. WT + 1 week after a 10 day dox treatment biological replicate 3
13. PRDM13-OE + 1 week after a 10 day dox treatment, biological replicate 3
14. WT + 3 weeks after a 10 day dox treatment, biological replicate 3
15. PRDM13-OE + 3 weeks after a 10 day dox treatment, biological replicate 3

Groups: 10 day dox, 1 and 2 mo
Technical replicate: 2
Target: Top blot: Robo3
Bottom blot: Gapdh – Loading Control
Imaging Type: ECL

Samples:
1. Ladder
2. WT + 1 week after a 10 day dox treatment, biological replicate 1
3. PRDM13-OE + 1 week after a 10 day dox treatment, biological replicate 1
4. WT + 3 weeks after a 10 day dox treatment, biological replicate 1
5. PRDM13-OE + 3 weeks after a 10 day dox treatment, biological replicate 1
6. Ladder
7. WT + 1 week after a 10 day dox treatment biological replicate 2
8. PRDM13-OE + 1 week after a 10 day dox treatment, biological replicate 2
9. WT + 3 weeks after a 10 day dox treatment, biological replicate 2
10. PRDM13-OE + 3 weeks after a 10 day dox treatment, biological replicate 2
11. Ladder
12. WT + 1 week after a 10 day dox treatment biological replicate 3
13. PRDM13-OE + 1 week after a 10 day dox treatment, biological replicate 3
14. WT + 3 weeks after a 10 day dox treatment, biological replicate 3
15. PRDM13-OE + 3 weeks after a 10 day dox treatment, biological replicate 3

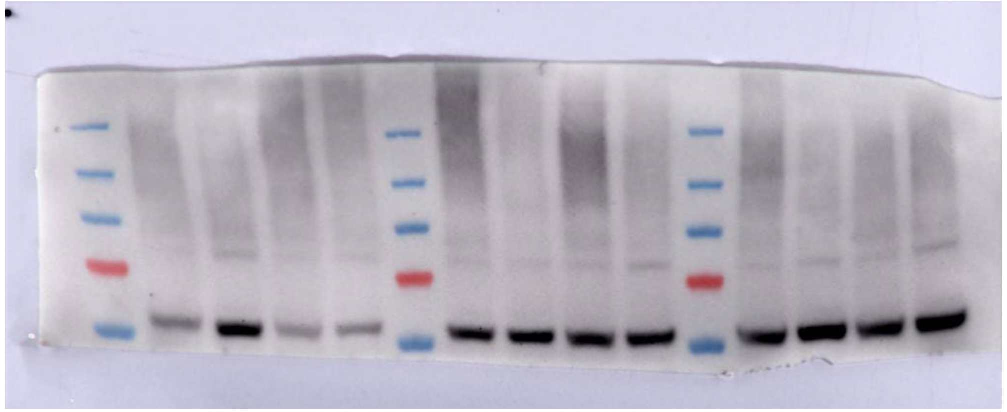

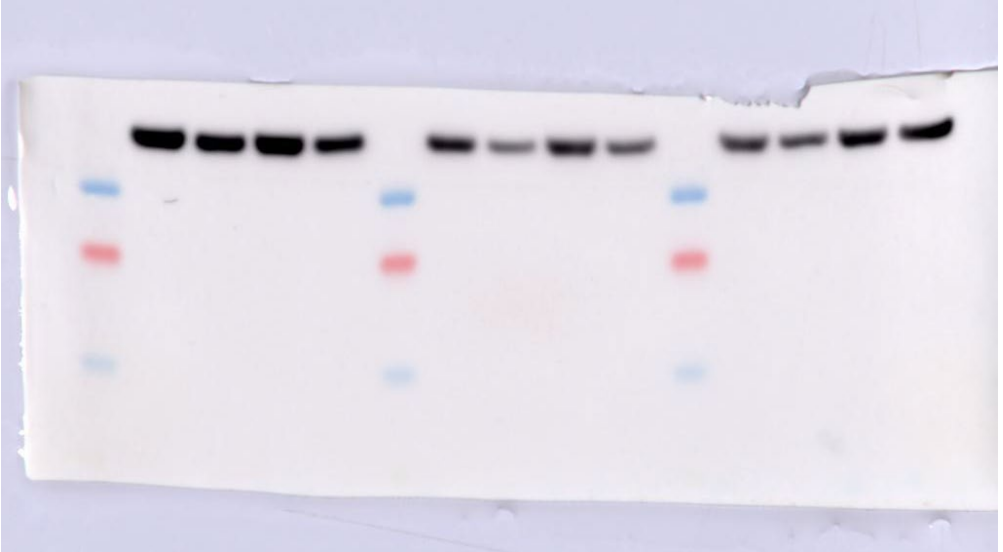

Groups: 3 days dox, on the 3rd day of dox
Technical replicate: 1
Target: Top blot: Vinculin – loading control
2nd blot: GAD65
3rd blot: Calretinin
Bottom blot: Calbindin
Imaging Type: Licor

Samples:
1. Ladder
2. WT + 3 days dox, biological replicate 1
3. PRDM13-OE + 3 days dox, biological replicate 1
4. WT + 3 days dox, biological replicate 2
5. PRDM13-OE + 3 days dox, biological replicate 2
6. WT + 3 days dox, biological replicate 3
7. PRDM13-OE + 3 days dox, biological replicate 3
8. WT + 3 days dox, biological replicate 4
9. PRDM13-OE + 3 days dox, biological replicate 4
10. WT + 3 days dox, biological replicate 5
11. PRDM13-OE + 3 days dox, biological replicate 5
12. EMPTY

**Did not include WT or PRDM13-OE biological replicate 5 in any analysis since I mis-cut the blots and cut off the bands.

**Grubb's outlier test showed that WT biological replicate 4 was a significant outlier, so it was removed from final analysis.

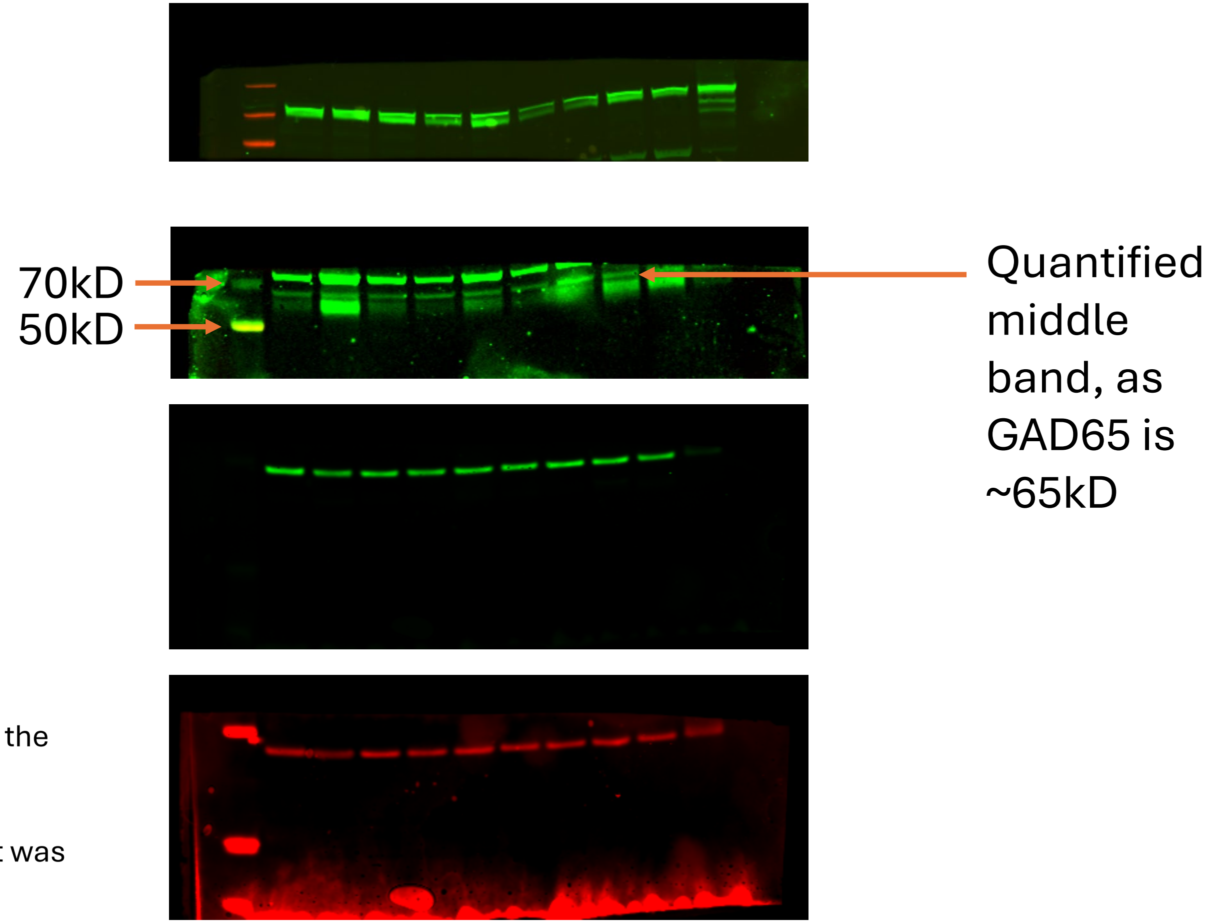

Groups: 3 days dox, on the 3rd day of dox
Technical replicate: 2
Target: Top blot: Vinculin – loading control
2nd blot: GAD65
3rd blot: Calretinin
Bottom blot: Calbindin
Imaging Type: Licor

Samples:
1. Ladder
2. WT + 3 days dox, biological replicate 1
3. PRDM13-OE + 3 days dox, biological replicate 1
4. WT + 3 days dox, biological replicate 2
5. PRDM13-OE + 3 days dox, biological replicate 2
6. WT + 3 days dox, biological replicate 3
7. PRDM13-OE + 3 days dox, biological replicate 3
8. WT + 3 days dox, biological replicate 4
9. PRDM13-OE + 3 days dox, biological replicate 4
10. WT + 3 days dox, biological replicate 5
11. PRDM13-OE + 3 days dox, biological replicate 5
12. EMPTY

**Did not include WT or PRDM13-OE biological replicate 5 in any analysis since I mis-cut the blots and cut off the bands on technical replicate 1.

**Grubb's outlier test showed that WT biological replicate 4 was a significant outlier, so it was removed from final analysis.

**Blot images converted to grayscale in final manuscript

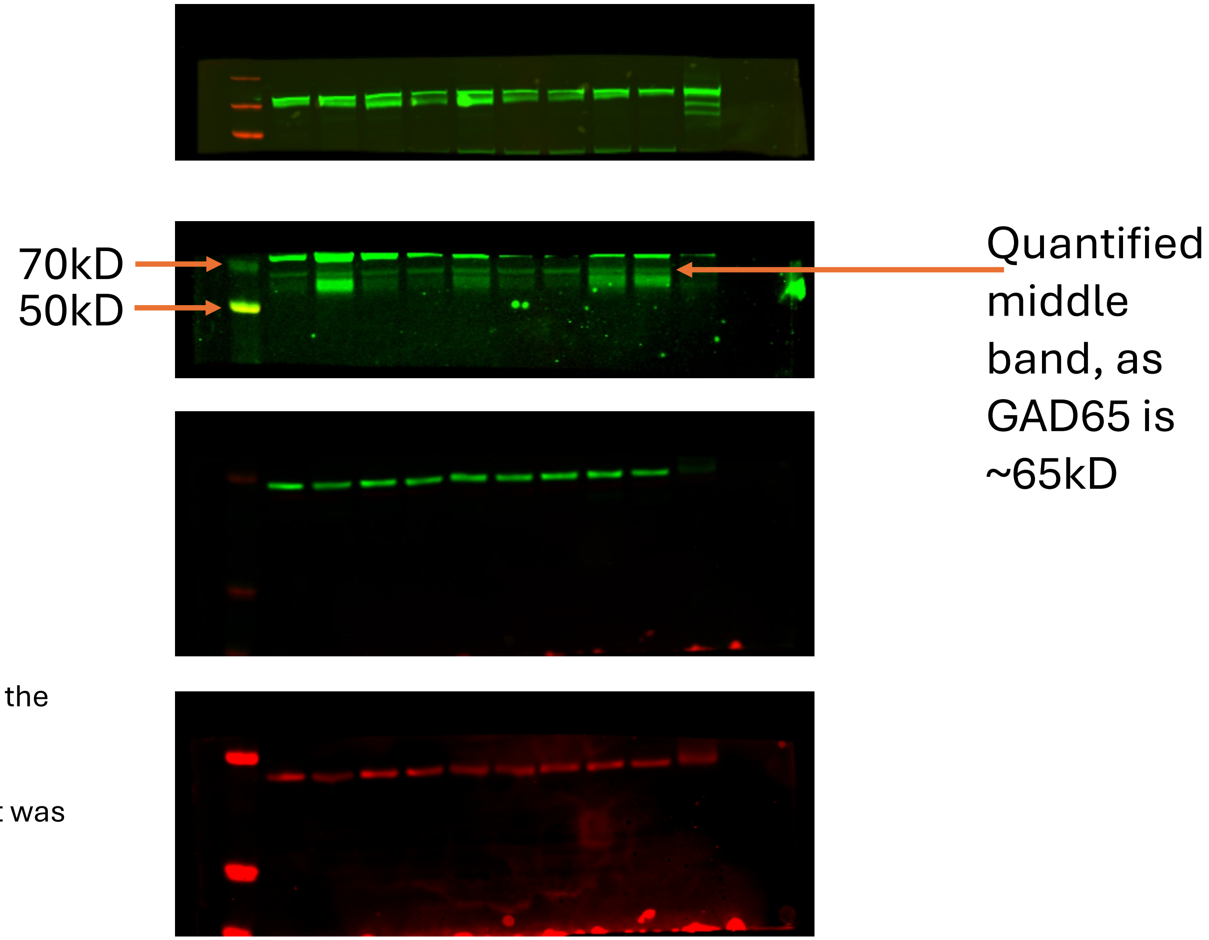

Supplement: Supplement 2 [file iovs-66-11-38_s002.pdf]
